# Understanding the Effects of Anode Catalyst Conductivity and Loading on Catalyst Layer Utilization and Performance for Anion Exchange Membrane Water Electrolysis

**DOI:** 10.1021/acscatal.4c02932

**Published:** 2024-07-03

**Authors:** Melissa E. Kreider, Haoran Yu, Luigi Osmieri, Makenzie R. Parimuha, Kimberly S. Reeves, Daniela H. Marin, Ryan T. Hannagan, Emily K. Volk, Thomas F. Jaramillo, James L. Young, Piotr Zelenay, Shaun M. Alia

**Affiliations:** † Chemistry and Nanoscience Center, National Renewable Energy Laboratory, Golden, Colorado 80401, United States; ‡ Center for Nanophase Materials Sciences, Oak Ridge National Laboratory, Oak Ridge, Tennessee 37830, United States; § Materials Physics and Applications Division, Los Alamos National Laboratory, Los Alamos, New Mexico 87545, United States; ∥ Department of Chemical Engineering, Stanford University, Stanford, California 94305, United States; ⊥ SUNCAT Center for Interface Science and Catalysis, SLAC National Accelerator Laboratory, Menlo Park, California 94025, United States; # Advanced Energy Systems Graduate Program, Colorado School of Mines, Golden, Colorado 80401, United States

**Keywords:** water electrolysis, oxygen evolution reaction, anion exchange membrane, electrocatalysis, catalyst layer

## Abstract

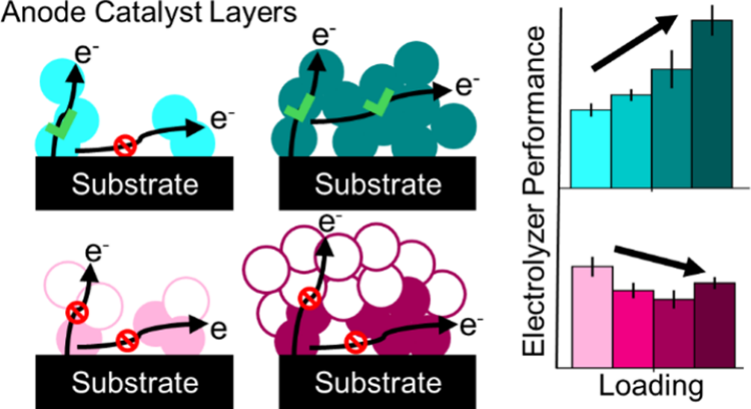

Anion exchange membrane water electrolysis (AEMWE) is a promising technology to produce hydrogen from low-cost, renewable power sources. Recently, the efficiency and durability of AEMWE have improved significantly due to advances in the anion exchange polymers and catalysts. To achieve performances and lifetimes competitive with proton exchange membrane or liquid alkaline electrolyzers, however, improvements in the integration of materials into the membrane electrode assembly (MEA) are needed. In particular, the integration of the oxygen evolution reaction (OER) catalyst, ionomer, and transport layer in the anode catalyst layer has significant impacts on catalyst utilization and voltage losses due to the transport of gases, hydroxide ions, and electrons within the anode. This study investigates the effects of the properties of the OER catalyst and the catalyst layer morphology on performance. Using cross-sectional electron microscopy and in-plane conductivity measurements for four PGM-free catalysts, we determine the catalyst layer thickness, uniformity, and electronic conductivity and further use a transmission line model to relate these properties to the catalyst layer resistance and utilization. We find that increased loading is beneficial for catalysts with high electronic conductivity and uniform catalyst layers, resulting in up to 55% increase in current density at 2 V due to decreased kinetic and catalyst layer resistance losses, while for catalysts with lower conductivity and/or less uniform catalyst layers, there is minimal impact. This work provides important insights into the role of catalyst layer properties beyond intrinsic catalyst activity in AEMWE performance.

## 1 Introduction

As the global economy seeks to decarbonize while maintaining current standards of living, it is critical to electrify and pursue carbon-free routes for the production of fuels and chemicals. Hydrogen is projected to play an important role in this transition as a fuel, chemical feedstock, and energy storage vector.^1,2^ In 2022, 99% of hydrogen was produced unabated from natural gas, coal, or as a byproduct of oil refining. Only 0.7% of the 95 million tons of H_2_ produced globally was considered low-emission, meaning that it was produced from fossil fuels with carbon capture or by using electricity.^3^ Of the low-emission H_2_ production approaches, water electrolysis offers the advantages of avoiding fossil fuels and coupling directly to renewable energy sources such as wind and solar. However, in the absence of emissions limitations or penalties, the cost of H_2_ production by electrolysis must be decreased significantly to compete with production from fossil fuels.^4−6^ Liquid alkaline (LA) and proton exchange membrane (PEM) electrolyzers have demonstrated success at the commercial scale, but they are limited by efficiency and cost, respectively. Anion exchange membrane electrolysis (AEMWE) is an emerging technology that seeks to combine the benefits of the alkaline environment of LAWE, which enables the use of inexpensive, earth-abundant catalysts and stack components, and the zero-gap architecture of PEMWE, which enables high efficiencies and dynamic operation.^7,8^ Research efforts have resulted in improved alkaline catalysts and membranes that have increased performance toward that of PEMWE, but significant challenges remain in terms of efficiency and durability to meet cost targets.^2,9−12^

To make AEMWE competitive with these more established electrolyzer technologies, both component-level and materials integration improvements must be made, particularly at the anode where the oxygen evolution reaction (OER) typically limits kinetic performance and durability.^7,13^ A significant advantage of AEMWE is the ability to replace platinum group metal (PGM) components of PEMWE with first-row transition metal-based materials. At the three-electrode liquid cell level, many promising catalysts have been developed for the OER, including first-row transition metal (e.g., Fe, Co, and Ni) oxides, X-ides (e.g., chalcogenides, pnictides, and carbides), and heterostructures.^14−17^ The activity and stability of these catalysts are closely related to the in situ oxidation processes that form active sites and determine the electronic conductivity.^18,19^ Research efforts have largely focused on increasing surface area, increasing bulk conductivity, and introducing dopants to tune active sites and control the oxidation processes.^15,17,20^ Because deployment of these materials in AEMWE is a more recent effort, however, it is not well understood which catalyst properties will be most important for performance in a device.^10,21^ In both PEMWE and AEMWE, the anode typically consists of a catalyst layer (CL), composed of OER catalyst particles and ion-conducting polymers, and a porous transport layer (PTL), which provides mechanical support to the membrane and transports electrons and evolved oxygen out of the catalyst layer. In PEMWE, significant research efforts have focused on optimizing the catalyst layer, including catalyst properties, ionomer, ionomer content, catalyst loading, and deposition methods. Improved PTL morphologies, compositions, and coatings have also been developed to decrease interfacial resistances and increase durability. These optimizations are important to maximize the number of accessible catalyst sites and minimize voltage losses due to the transport of oxygen (bubbles), electrons, and ions within the anode.^22−24^ The through-plane conduction of ions from the membrane to the catalyst active sites and in- and through-plane conduction of electrons from catalyst active sites to the PTL are encapsulated in the term catalyst layer resistance (*R*_CL_), which impacts the effective overpotential within the catalyst layer and the catalyst utilization.^25−27^ Analyses have been developed to determine the *R*_CL_ from electrochemical impedance spectroscopy (EIS) using a transmission line model and to calculate the corresponding voltage loss, allowing for the correlation of catalyst layer properties to their effects on performance.^25^ For PEMWE anodes, in-plane electronic conductivity has been found to be limiting, particularly at low catalyst loadings. One study found that bubble formation occurred only at locations where the catalyst layer made direct contact with the PTL, indicating that electron conduction across the catalyst layer was limiting.^28^ Several electrode factors impact this effective in-plane conductivity, including the intrinsic conductivity of the catalyst particles,^29^ the thickness and uniformity of the catalyst layer,^30,31^ the morphology of the PTL,^30^ and the quality of the PTL-catalyst layer interface.^32^ These electrode design trade-offs are challenging for PEMWE since homogeneous catalyst layers are difficult to achieve at the low iridium loadings needed to meet cost targets.^31,33^

While AEMWE does not have the same limitations of catalyst cost, the properties of the catalyst, ionomer, and PTL are currently inferior to those of PEMWE.^34^ First, the transition metal oxides typically used as alkaline OER catalysts have orders of magnitude lower electronic conductivity than IrO_2_, which can lead to large ohmic losses.^35^ A study comparing OER catalysts in RDE and AEMWE found that catalyst performance in AEMWE was more strongly correlated to the conductivity of a catalyst than its intrinsic OER activity.^36^ Furthermore, the catalyst particle/aggregate sizes are typically too large for ultrasonic spraying, a common membrane electrode assembly (MEA) manufacturing technique used for PEMWE.^37^ The MEAs for AEMWE, therefore, often have nonuniform catalyst layers with large catalyst agglomerates and inhomogeneous distribution of ionomer, which can lead to mechanical instability of the catalyst layer, decrease catalyst site accessibility, and increase catalyst layer resistances. While the anion exchange ionomers can have comparable ionic conductivity to Nafion, they are known to degrade rapidly under the oxidative conditions at the anode, causing particle detachment as well as decreased ionic conductivity, particularly in pure water operation.^38−42^ Ionic conductivity is a lesser concern in high conductivity supporting electrolytes such as 1 M KOH, so electronic resistances are expected to dominate the *R*_CL_ losses.^43^ Finally, the PTL materials available for AEMWE typically have high porosities and large pores, resulting in low material density at the interface with the catalyst layer. The distance between the PTL contact points can be equal to or greater than the catalyst layer thickness, increasing the reliance on in-plane electronic conductivity and decreasing the number of usable catalyst sites.^44−46^ To translate the advances made in catalyst and polymer development to overall device performance, it is necessary to improve their integration into the catalyst layer.

In this work, we compare the AEMWE performance of four OER catalysts in 1 M KOH supporting electrolyte: Ni–Fe aerogel and commercial NiFe_2_O_4_, Co, and Co_3_O_4_. By comparing catalysts with similar active site composition, we aim to understand how their material properties (e.g., conductivity, surface area, and crystallinity) affect the nature of the catalyst layer, site accessibility, and site utilization. We further use a loading study to probe how changes in catalyst thickness and morphology lead to performance improvements for some catalysts, while having minimal impacts on others. Overall, this work provides insight into catalyst layer design strategies to optimize anode performance in AEMWE.

## 2 Methods

### 2.1 Catalyst Materials

Commercial catalysts were used without further treatment: NiFe_2_O_4_ (US Research Nanomaterials Inc., 98%), Co_3_O_4_ (US Research Nanomaterials Inc., 99%), Co (core)/CoO_*x*_ (shell, 2 nm) (Alfa Aesar, 99.8%), and Pt/C (47% Pt, TKK TEC10E50E). The Ni_8_Fe catalyst was synthesized from NiCl_2_·6H_2_O (Fisher Scientific), FeCl_2_·4H_2_O (Sigma-Aldrich), poly(acrylic acid) (Sigma-Aldrich, MW 450,000), and ethanol (Sigma-Aldrich, 200-proof), as described previously.^20^

### 2.2 Physical and Chemical Characterization

Brunauer–Emmett–Teller (BET) surface areas were calculated from N_2_ physisorption measurements at 77 K using a Quantachrome Autosorb iQ. X-ray diffraction (XRD) patterns were collected using a Bruker D8 Discover with Cu Kα radiation (λ = 0.15406 nm) in the 2θ range between 13.5 and 88°. X-ray photoelectron spectroscopy (XPS) characterization of anode catalyst layers was conducted using a Phi Versaprobe 4 with monochromatized Al Kα radiation (1486 eV). The samples were neutralized using an Ar ion gun and an electron flood gun. The high-resolution spectra were collected at high power (100 W, 20 kV), with a spot size of 100 × 100 μm^2^ and a dwell time of 20 ms. The pass energy was 55 eV for the Co, Fe, and Ni 2p spectra and 13 eV for the C, N, O, and F 1s spectra. The spectra were calibrated by shifting the C–C 1s peak to 284.8 eV and analyzed using CasaXPS software^47^ according to the literature.^48^ Catalyst loadings on the anode and cathode were taken as the average of 3 measurements with 30 s exposure using X-ray fluorescence (Fischer XDV-SDD XRF).

Scanning transmission electron microscopy (STEM) was used to characterize catalyst materials before and after MEA testing. A small portion of PTL was sonicated in isopropanol to disperse the catalyst material. Then, the catalyst dispersion was drop-casted onto a transmission electron microscopy (TEM) grid for imaging. High-angle annular dark-field (HAADF) and energy-dispersive X-ray spectrum (EDS) images were recorded using a JEM-ARM200F “NEOARM” analytical electron microscope (JEOL Ltd.) operated at 200 kV, equipped with a dual windowless silicon-drift detector (SDD) each with a 100 mm^2^ active area. EDS maps were processed using JEOL Analysis Station (JEOL Ltd.) software. Anode cross sections were imaged using a scanning electron microscope (SEM) with a backscattered electron (BSE) detector on a Hitachi S4800 operated at 5 kV. Specimens were prepared by embedding a small piece of catalyst-coated PTL in an epoxy (mixed with graphite to enhance electron conductivity). The epoxy block was polished to expose the anode catalyst layer cross section for imaging. Anode catalyst layer thicknesses were approximated from cross-sectional SEM images, averaging from 35 locations along the catalyst layer using ImageJ.

Ex situ, in-plane conductivity measurements of the catalyst layers at ∼0.6 mg/cm^2^ loading sprayed on Nafion 115 membrane (chosen as an electrically insulating substrate for the measurements) were conducted with a modified four-point probe technique, which uses parallel thin gold ribbons (18.5 × 250 μm^2^) instead of zero-dimensional probe tips. A slow voltage scan rate of 2 mV/s was used to isolate electronic conductivity. An adjusted mathematical model and correction factor were developed to achieve *R*_sheet_ values in Ω/square. Results are reported with ±10% uncertainty.

### 2.3 Electrochemical Characterization

Water electrolysis performance was tested in single-cell, membrane electrode assemblies (MEAs). The cathodes consisted of a commercial Pt/C catalyst deposited on a 5 cm^2^ carbon paper gas diffusion layer (Fuel Cell Earth, MGL280, 80280-40) using an airbrush spraying method (vacuum plate, 80 °C). A typical cathode ink was composed of 50 mg Pt/C, 1.7 mL *n*-propanol (Sigma-Aldrich, OmniSolv, high-performance liquid chromatography (HPLC) grade), 2.3 mL deionized water (Milli-Q; ≥18.2 MΩ cm resistivity), and 0.43 g ionomer (Versogen, PiperION-A TP-85, 5 wt % in ethanol), targeting a 0.3 mg_Pt_/cm^2^ loading and 30 wt % ionomer-to-(ionomer + catalyst) ratio. The anodes consisted of the Ni_8_Fe, NiFe_2_O_4_, Co@CoO_*x*_, and Co_3_O_4_ catalysts airbrush sprayed onto 5 cm^2^ Ni porous transport layers (Bekaert, BEKIPOR 2Ni 18–0.25). The anode ink formulations varied based on the catalyst (metal %) and target loading but generally were composed of catalyst, 10 vol % deionized water, 90 vol % n-propanol, and ionomer (PiperION-A TP-85, target of 30 wt % ionomer-to-(ionomer + catalyst) ratio). Anode loadings were targeted to 0.3, 0.6, 0.9, and 2.5 mg_TM_/cm^2^ by adjusting the total ink volume.

Prior to assembly, the membranes (Versogen, PiperION-A TP-85, 80 μm) were ion-exchanged from carbonate to hydroxide form in 3 M KOH (EMD Millipore, Emsure grade) for 48 h, and the electrodes were ion-exchanged in 0.5 M KOH for 30 min to shorten the needed cell break-in time. The 25 cm^2^ hardware was custom-made and consisted of anodized Al (Fuel Cell Technologies) or stainless steel (316L) end plates, Au-coated current collectors (Cu plated with 50–100 μm Ni and 30 μm Au), and Ni triple-serpentine flow fields (Ni 200). Poly(tetrafluoroethylene) (PTFE) gaskets (254 μm at the cathode, 280 μm at the anode, no edge protection) were used to achieve approximately 20% compression of the MEA at 4.5 N m torque.

All MEA testing was conducted at 80 °C with 1 M KOH supporting electrolyte supplied to the anode and the cathode at 50 mL/min each. The electrolyte headspace was purged with N_2_ to prevent carbonation of the electrolyte and membrane. Electrochemical measurements were performed using an Autolab PGSTAT302N potentiostat with a 20 A booster (Eco Chemie, Metrohm Autolab). The MEAs were tested with the following potentiostatic protocol: (a) polarization curve with 20 s holds at 2.00, 1.90, 1.80, 1.70, 1.65, 1.60, 1.55, 1.525, 1.50, 1.48, 1.46, 1.44, 1.42, 1.41, and 1.40 V, and the reverse back to 2.00 V; (b) 2 h cell conditioning hold at 2 V; (c) polarization curve with 2 min holds at the voltages from step (a); (d) electrochemical impedance spectroscopy (EIS), with AC amplitude of 10% of the voltage value from 18 kHz to 1 Hz at non-Faradaic voltages of 1.25, 1.30, and 1.35 V and the voltages from step (a); and (e) cyclic voltammetry (CV) at scan rates of 20, 50, and 100 mV/s from 0 to 1.4 V. The high-frequency resistance (HFR) was determined at every voltage via interpolation of the high-frequency region in the EIS spectra. Catalyst layer resistance was calculated from the non-Faradaic EIS using linear fitting and a transmission line model.^25,49^ While a Tafel slope cannot technically be determined from this two-electrode measurement, the slope of the voltage–logarithm (current) relationship provides insight into the kinetics of the overall system. These slopes, denoted as *V*–log(*I*) slope, were calculated using HFR-free voltages in the current range of 5–50 mA/cm^2^. The equilibrium cell potential was corrected for the atmospheric pressure of the test site (82.2 kPa) and elevated testing temperature (80 °C); details of the calculation are given in the Supporting Information (SI).^50,51^

## 3 Results and Discussion

### 3.1 Ni–Fe Catalysts: Ni_8_Fe Aerogel and NiFe_2_O_4_

Nickel–iron oxide catalysts are among the most active alkaline OER catalysts in three-electrode tests.^52^ Because the starting oxide materials are often insulators or semiconductors, the potential at which the catalyst undergoes oxidation to more conductive oxyhydroxide phases often determines the onset potential.^53,54^ Thus, the composition of the catalyst, such as the Ni-to-Fe ratio or the presence of other dopant metals, is important both to tune the binding energy for oxygen intermediates and to lower the redox potential.^55^ However, it is not yet clear whether these same in situ processes and structure–activity relationships determine performance in the unique microenvironment of the single-cell AEMWE. In particular, the importance of catalyst surface area and electrical conductivity in MEAs, specifically in supporting electrolyte, is not well understood. These properties have obvious corollaries to site availability/utilization and catalyst layer resistance, and thus electrochemical analysis tools exist to separate out these effects.

In this study, we compare a Ni_8_Fe aerogel catalyst,^20^ whose composition and crystallinity have been optimized for OER activity, and a commercial NiFe_2_O_4_ catalyst (USRM). The aerogel performance was found to be optimized with a high Ni-to-Fe ratio and a low annealing temperature, which resulted in an amorphous structure (Figure S1) and a high BET surface area of 382 m^2^/g.^20^ High-angle annular dark-field-scanning transmission electron microscopy (HAADF-STEM) images of agglomerates and single nanoparticles show small, randomly ordered crystallites (Figure S2). In contrast, the commercial NiFe_2_O_4_ material has a highly ordered spinel structure (Figure S1), larger crystallites (Figure S2), and a lower BET surface area of 77 m^2^/g.^21^ For both materials, energy-dispersive X-ray spectroscopy (EDS) elemental maps indicate a homogeneous distribution of Ni, Fe, and O throughout the particles (Figure S2), and the nominal Ni-to-Fe ratios are confirmed as 7.4 for Ni_8_Fe and 0.54 for NiFe_2_O_4_. Both catalysts are significantly oxidized, with O-to-metal ratios of ∼3. X-ray photoelectron spectroscopy (XPS) fits confirm Ni^2+^ and Fe^3+^ average oxidation states in NiFe_2_O_4_, while the Ni_8_Fe is fit to a combination of Ni(OH)_2_ and NiOOH (Figure S3). Finally, in-plane sheet resistance measurements of the two catalysts, sprayed in catalyst layers at ∼0.6 mg_TM_/cm^2^ loading and 30 wt % ionomer on Nafion 115 membrane, show significant differences, with 44 kΩ/square and 131 kΩ/square for Ni_8_Fe and NiFe_2_O_4_, respectively (Figure S4). There are limitations to how this metric can be used to understand in situ, in-plane conductivity because the catalyst layer is deposited on a membrane instead of the PTL, and the measurement is performed ex-situ, so it cannot capture conductivity changes that occur in a hydrated environment due to redox transitions.^54^ However, it allows for a qualitative comparison of conductivity and provides insight into the combined effects of intrinsic catalyst conductivity and catalyst layer uniformity. In the future, in situ measurements of in-plane conductivity as a function of cell voltage would provide significant insights into the dynamics of catalyst layer conductivity.

**Figure 1:**
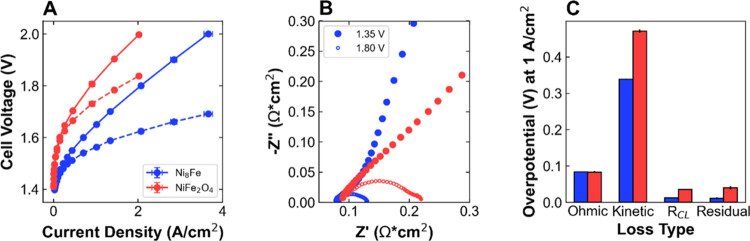
Performance and voltage loss breakdown: Ni_8_Fe and NiFe_2_O_4_ anode catalysts. (A) Polarization curves with (dashed lines) and without (solid lines) HFR-correction, (B) Nyquist plot of electrochemical impedance spectra at 1.35 V (solid circles) and 1.80 V (open circles), and (C) summary of the distribution of overpotential between the different voltage losses at 1 A/cm^2^ for Ni_8_Fe (blue) and NiFe_2_O_4_ (red). Performance data is reported in triplicate; EIS spectra are representative of the average behavior. Details of the voltage loss breakdown analysis are shown in Figure S5.

Although they do not necessarily have identical active sites, since the Ni-to-Fe ratio may tune the binding energetics of Ni sites for OER intermediates, comparing Ni_8_Fe and NiFe_2_O_4_ can provide insight into the effects of catalyst conductivity, crystallinity, surface area, and particle morphology in an AEMWE. At a total anode metal loading of 0.6 mg/cm^2^, Ni_8_Fe had a significantly higher performance, reaching 1 A/cm^2^ at 1.648 V compared to 1.823 V for NiFe_2_O_4_ (Figure 1A). Improved performance is also observed at 2 V, reaching a maximum current density of 3.66 A/cm^2^ compared to 2.02 A/cm^2^. The high-frequency resistance (HFR) is similar for both catalysts at ∼80 mΩ cm^2^, resulting in similar ohmic losses (Figures 1B,C and S5), but the charge transfer resistance at 1.8 V is much larger for NiFe_2_O_4_. The HFR-corrected voltage at 1 A/cm^2^ for the Ni_8_Fe aerogel is 1.563 V, which is within 22 mV of a reference PEMWE cell.^56^ The Ni_8_Fe further shows a low *V*–log(*I*) slope of 73 mV/decade in the kinetic region (defined as between 5 and 50 mA/cm^2^ current density) compared to 90 mV/decade for NiFe_2_O_4_, as well as a higher *V*–log(*I*) intercept (analogous to an exchange current density) of 16 vs 5 μA/cm^2^, resulting in much lower kinetic overpotentials for the Ni_8_Fe catalyst (Figure S5). While these single-cell electrochemical measurements correspond to the combined behavior of the anode and the cathode, we assume that the OER is rate-limiting and controls the kinetics. The differences in kinetic parameters between the two anodes may be due to both differences in intrinsic catalyst activity on distinct active sites and catalyst layer resistances that decrease site utilization and increase the effective *V*–log(*I*) slope.^25^ The electrochemically accessible surface area, calculated from cyclic voltammograms or electrochemical impedance spectra (EIS), is a useful metric for understanding catalyst site accessibility. However, there are challenges to this calculation,^57,58^ particularly due to overlapping Faradaic processes and contributions of the PTL to the electrochemical behavior. Figure S6 shows that the Ni PTL is an active anode without a catalyst layer, reaching 1.32 A/cm^2^ at 2 V, and cyclic voltammograms show similar redox features and amounts of current for Ni_8_Fe, NiFe_2_O_4_, and bare Ni PTL in the 1–1.4 V potential window, complicating efforts to extract catalyst-specific surface area information. Between 0 and 0.8 V, however, the catalyst-coated PTLs show much larger currents and redox features than the bare PTL, indicating that differences in this potential window may be attributed to the catalyst layer. However, the Faradaic processes throughout the voltammograms prevent accurate determination of capacitance or surface area.

Impedance analysis can provide further insight into the electrochemical behavior, including surface area and catalyst layer resistance. Recent studies have shown that EIS can be used to determine the capacitance at both Faradaic and non-Faradaic voltages.^57,58^ Fitting the EIS spectra at 1.35 V (Figure 1B), a non-Faradaic voltage where redox activity has been observed for both the catalyst-coated and bare PTLs, using a transmission line model,^49^ 2.5× and 5.5× larger capacitance is found for Ni_8_Fe than NiFe_2_O_4_ and Ni PTL, respectively. The *R*_CL_ can also be calculated from these EIS spectra using the transmission line curve fit or a linear intercept fit.^25^ While the trends between the two methods are found to be consistent for these samples, the transmission line model overestimates the *R*_CL_, perhaps due to the curved shape of the spectra, leading to negative residual losses (Figure S7). Therefore, the *R*_CL_ values calculated from the linear intercept fit are used throughout this discussion. The fits reveal significant differences in the *R*_CL_ between the catalysts. As discussed previously, the *R*_CL_ is indicative of resistance to through-plane ionic and in- or through-plane electronic transport within the catalyst layer. The *R*_CL_ is affected by properties of the electrode architecture such as the distance between PTL fibers, the amount of ionomer, and the presence of supporting electrolyte, as well as the electronic resistivity of catalyst particles. Comparing the MEAs that utilize the same catalyst loading, PTL, supporting electrolyte, and ionomer type and content, the differences in *R*_CL_, 100 and 580 mΩ cm^2^ for Ni_8_Fe and NiFe_2_O_4_, respectively, are attributable to differences in catalyst layer properties, such as catalyst conductivity, particle/agglomerate size, and catalyst layer morphology. These *R*_CL_ values are significantly higher than those observed for well-optimized PEMWEs,^25^ and thus, this is an important source of overpotential to investigate and optimize. The *R*_CL_ overpotential is much higher for NiFe_2_O_4_, which also results in a significant decrease in catalyst utilization (Figure S5). Finally, the residual loss, which may be due to bubble formation or gas transport, is also higher for NiFe_2_O_4_ (Figures 1C and S5), indicating further nonidealities of the catalyst layer. Overall, Ni_8_Fe significantly outperforms NiFe_2_O_4_, primarily due to lower kinetic, catalyst layer resistance, and residual transport losses.

### 3.2 Co Catalysts: Co@CoO_*x*_ and Co_3_O_4_

Cobalt-based catalysts have also been intensely studied for the alkaline OER.^52,59^ Like NiFe-based catalysts, the active phase is generally considered to be a Co oxyhydroxide, and conversion to higher Co oxidation states (≥3+) is understood to be important for activity.^18^ While spinel cobalt oxides are commonly used as OER catalysts, they are generally more insulating and more resistant to in situ oxidation than comparable defected or doped structures.^36,59,60^ This has driven interest in heterostructure nanoparticle catalysts, with a conductive, nonoxide core and oxidized surface, but an understanding of the behavior of these materials in AEMWE is limited.^19^ In this work, we study commercial Co and Co_3_O_4_ nanoparticles, which allows for a direct comparison between catalysts with major differences only in degree of oxidation and conductivity. Both have roughly spherical particles with a size of ∼25–30 nm, but Co_3_O_4_ has a much larger BET surface area^21^ of 37 m^2^/g compared to 14 m^2^/g for Co. Co_3_O_4_ shows the expected spinel structure (Figure S8), single crystal-like uniformity (Figure S9), and an even distribution of Co and O by STEM-EDS (Figure S10). HAADF images and STEM-EDS maps show that the Co particles have a metallic core and few nanometer-thick oxidized shells (Figures S9 and S10); this catalyst will, therefore, be denoted as Co@CoO_*x*_. Possible changes to the metallic core during testing will be discussed in Section 3.4. The shell results in significant oxidized character in XPS (Figure S11) and the presence of diffraction peaks corresponding to an oxidized Co species in addition to face-centered cubic (fcc) Co in the XRD (Figure S8). Comparing catalyst layers with anode Co loading of ∼0.5 mg/cm^2^ sprayed on Nafion membrane, there is also a difference in in-plane sheet resistance, with 44 and 96 kΩ/square for Co@CoO_*x*_ and Co_3_O_4_, respectively (Figure S4), indicating the impact of the metallic core on the overall conductivity.

**Figure 2:**
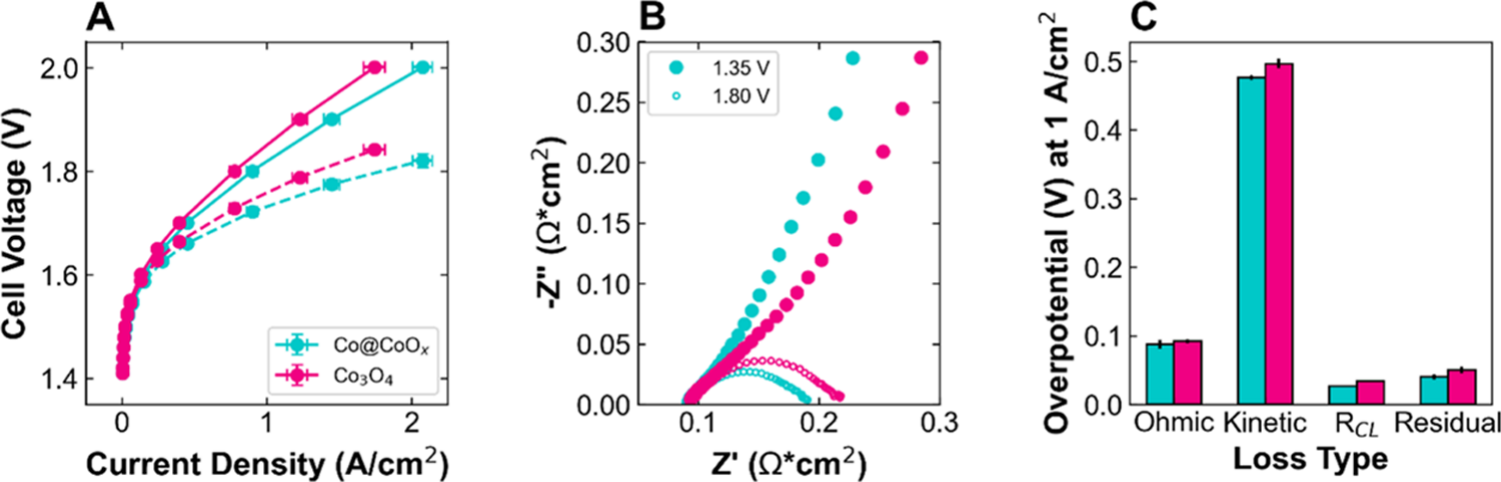
Performance and voltage loss breakdown: Co@CoO_*x*_ and Co_3_O_4_ anode catalysts. (A) Polarization curves with (dashed lines) and without (solid lines) HFR-correction, (B) Nyquist plot of electrochemical impedance spectra at 1.35 V (solid circles) and 1.80 V (open circles), and (C) summary of the distribution of overpotential between the different voltage losses at 1 A/cm^2^ for Co@CoO_*x*_ (teal) and Co_3_O_4_ (pink). Performance data is reported in triplicate; EIS spectra are representative of the average behavior. Details of the voltage loss breakdown analysis are shown in Figure S12.

Due to the similarities of the active sites, the study of Co@CoO_*x*_ and Co_3_O_4_ allows for a direct comparison of the effects of anode catalyst oxidation, crystallinity, and conductivity in AEMWE. At a total anode metal loading of 0.6 mg/cm^2^, Co@CoO_*x*_ had a significantly higher performance, reaching 1 A/cm^2^ at 1.739 V compared to 1.758 V (HFR-free) for Co_3_O_4_ (Figure 2A). Improved performance is also observed at 2 V, reaching a maximum current density of 2.10 A/cm^2^ compared to 1.74 A/cm^2^. The EIS at 1.8 V shows slightly lower HFR and charge transfer resistance values for Co@CoO_*x*_ (Figure 2B). The HFR and associated ohmic losses are slightly higher for Co_3_O_4_, particularly at higher current densities (Figures 2C and S12); the possible relationship between HFR and low catalyst conductivity will be discussed in later sections. While Co@CoO_*x*_ has a slightly lower *V*–log(*I*) intercept than Co_3_O_4_ (7 vs 10 μA/cm^2^), the lower *V*–log(*I*) slope of 96 mV/dec compared to 102 mV/dec results in a slightly lower kinetic overpotential, particularly at high current densities (Figures 2C and S12). These small differences may again be due to differences in the in situ active site or catalyst layer properties that affect utilization. As was observed for the NiFe catalysts, many of the features in the CV are the same as those present for the bare Ni PTL (Figure S13). Fitting the EIS spectra at the non-Faradaic voltage of 1.35 V with a transmission line model, the capacitance for Co_3_O_4_ is found to be 1.25× and 3× larger than Co@CoO_*x*_ and the Ni PTL, respectively. This is a smaller difference in capacitance than in BET surface area, indicating that not all of the Co_3_O_4_ surface area is electrochemically accessible. This may relate to differences in the catalyst layer morphology and porosity, which will be discussed in the next section. Transmission line modeling also shows significant differences in the *R*_CL_ between the MEAs (Figure 2B). The differences in *R*_CL_, 320 and 455 mΩ cm^2^ for Co@CoO_*x*_ and Co_3_O_4_, respectively, are likely due to differences in catalyst layer properties, especially catalyst conductivity, which contributes to catalyst layer electronic resistance. The *R*_CL_ overpotential is higher for Co_3_O_4_, which also results in a significant decrease in catalyst utilization (Figure S12). Finally, the residual transport loss is similar for the two catalysts (Figure 2C). Overall, Co@CoO_*x*_ outperforms Co_3_O_4_, due to differences in ohmic, kinetic, and catalyst layer resistance losses.

### 3.3 Anode Loading Effects

**Figure 3:**
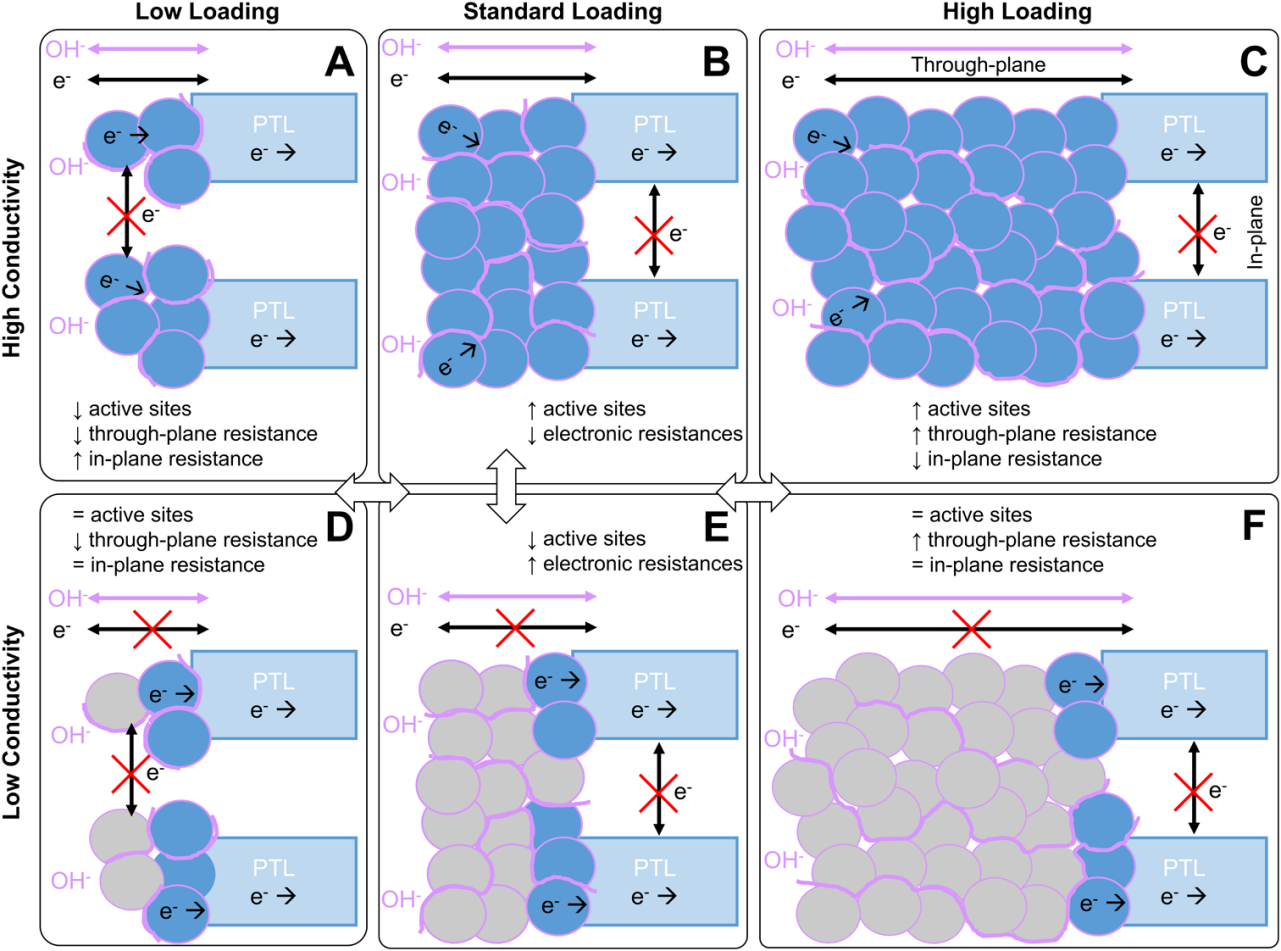
Schematic illustrating the effects of anode catalyst layer loading and electronic conductivity on the active site accessibility, through-plane ionic and electronic resistances, and in-plane electronic resistance. Cross-sectional view of catalyst layer on PTL substrate (membrane on left, not shown). (A–C) High electronic conductivity catalyst at low, standard, and high loadings. (D–F) Low electronic conductivity catalyst at low, standard, and high loadings. Blue circle = accessible catalyst particles; gray circle = inaccessible catalyst particles; light blue rectangle = PTL fibers; purple line = example of ionomer network/supporting electrolyte providing OH^–^ transport; black arrow = electron transport; and purple arrow = OH^–^ transport.

There are a few possible effects that anode loading can have on performance and resistances through the catalyst layer, depending on the electronic conductivity of the catalyst as well as the morphology of the catalyst layer, as illustrated in the simplified schematic of the catalyst layer in Figure 3. First, with increased loading, we may expect to have more active sites available for the reaction as well as to form a more continuous, uniform catalyst layer that can improve the in-plane electronic resistances related to the large gaps between the PTL fibers. Comparing Figure 3A,B, it is clear that the increase in loading allows for improved in-plane electron transport, thereby effectively increasing the number of active sites. For a low conductivity system, however, the increase in loading (Figure 3D,E) does not lead to a significant increase in active sites or a decrease in in-plane conductivity, since electron transport is limited by the insulating particles. Further increases in loading, as in Figure 3E,F, would be expected to have a negative effect on performance, as there will be an increase in through-plane electronic and ionic resistances to increase with increased loading or catalyst layer thickness. For high loading with a high electronic conductivity catalyst (Figure 3C), this increased through-plane resistance may be balanced or overcome by the effects of having more active sites and improved in-plane conductivity, such that overall performance may improve or be unchanged at higher loadings. Overall, we can expect the effects of loading to vary based on several factors, including catalyst electronic conductivity, catalyst layer morphology and uniformity, ionomer/ion transport networks, and PTL porosity and pore size. These effects may show up in a variety of places within the voltage losses: through-plane electronic resistance affects the HFR or ohmic losses; the number of active sites impacts the kinetics; as previously discussed, electronic and ionic resistances affect the catalyst layer resistance; and gas/liquid transport determines the residual or mass transport losses. By conducting a loading study with the four catalysts discussed to this point, which vary in particle surface area, conductivity, and catalyst layer morphology, we can gain insight into the dominant effects and optimal catalyst layer construction.

**Figure 4:**
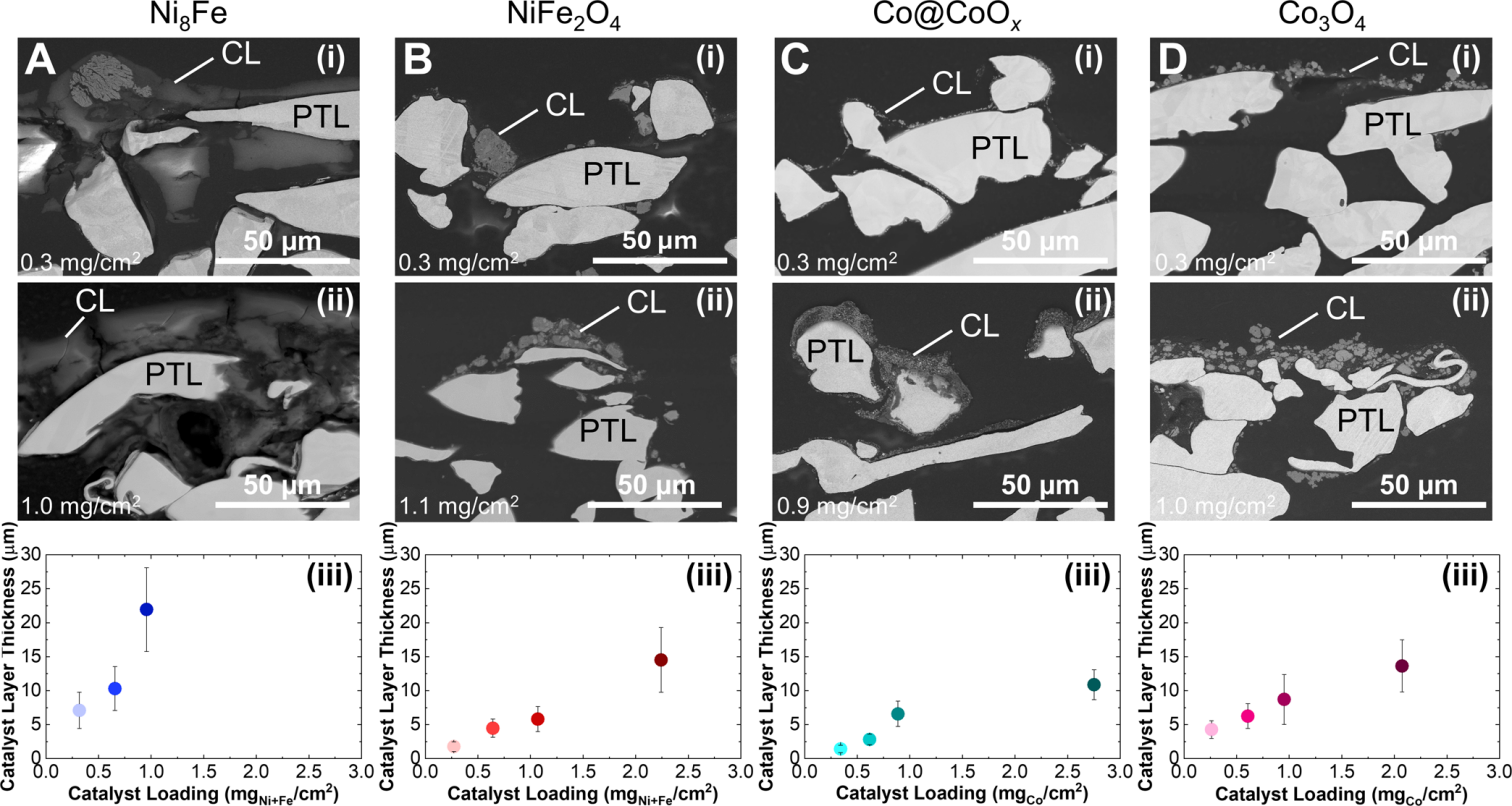
Cross-sectional SEM images of anode catalyst layers on Ni PTL. (A) Ni_8_Fe, (B) NiFe_2_O_4_, (C) Co@CoO_*x*_, and (D) Co_3_O_4_ at loadings of (i) 0.3 mg/cm^2^ and (ii) 1.0 mg/cm^2^. (iii) Summary of catalyst layer thickness as a function of loading for Ni_8_Fe (blue), NiFe_2_O_4_ (red), Co@CoO_*x*_ (teal), and Co_3_O_4_ (pink). Thicknesses are reported as an average of 35 measurements from the images. The catalyst layer and PTL fibers are labeled in the cross-sectional images.

Using four loadings of approximately 0.3, 0.6, 1.0, and 2.2 mg/cm^2^ (total metal basis), we can explore a range of catalyst layer thicknesses and PTL coverages. Figure 4 shows cross-sectional SEM images at loadings of 0.3 and 1.0 mg/cm^2^ as well as calculated catalyst layer thicknesses. Cross-sectional images for all loadings are shown in Figure S14. Compared at 0.3 mg/cm^2^, there is a significant spread in the average catalyst layer thickness: 1.4, 1.8, 4.3, and 7.1 μm, which increases to 6.6, 5.8, 8.7, and 21.9 μm at 1 mg/cm^2^ for Co@CoO_*x*_, NiFe_2_O_4_, Co_3_O_4_, and Ni_8_Fe, respectively. In addition to being thicker, the higher-loading catalyst layers are more homogeneous, cover more of the PTL fibers, and have improved continuity across the gaps between the PTL fibers. At all loadings, the Ni_8_Fe catalyst layers have the best homogeneity and PTL coverage (Figures 4A and S15), while the commercial catalysts tend to form isolated clusters of catalyst. In particular, large particle clusters within the catalyst layer are visible for NiFe_2_O_4_ and Co_3_O_4_ at all loadings (Figure 4B,C), while Co@CoO_*x*_ has smaller clusters and their dispersion within the catalyst layer improves with increased loading (Figure 4D). At 0.3 mg/cm^2^, there are large gaps between the commercial catalyst particles, both along and between fibers. Even at high loadings, the distance between PTL fibers (∼20 μm) is larger than the thickness of most catalyst layers, supporting the hypothesis that in-plane electronic conductivity will be more limiting than through-plane. In addition to the intrinsic catalyst properties (e.g., conductivity, OER activity), these morphological properties of the catalyst layer can have significant impacts on anode performance. As discussed previously, the large contribution of the Ni PTL to the redox features and capacitance makes this data difficult to interpret (Figure S16). For Ni_8_Fe, EIS fitting at 1.35 V and cyclic voltammetry indicates that there is a ∼20% increase in surface area with the increase in loading from 0.3 to 0.6 mg/cm^2^. However, there is no further increase at 1 mg/cm^2^. Co@CoO_*x*_ has no significant changes to either measure of surface area with loading, while both Co_3_O_4_ and NiFe_2_O_4_ show small decreases at higher loading. While the CVs should not be overinterpreted, these different effects of loading on capacitive behavior may reflect differences in the catalyst layer morphology, porosity, and PTL coverage.

**Figure 5:**
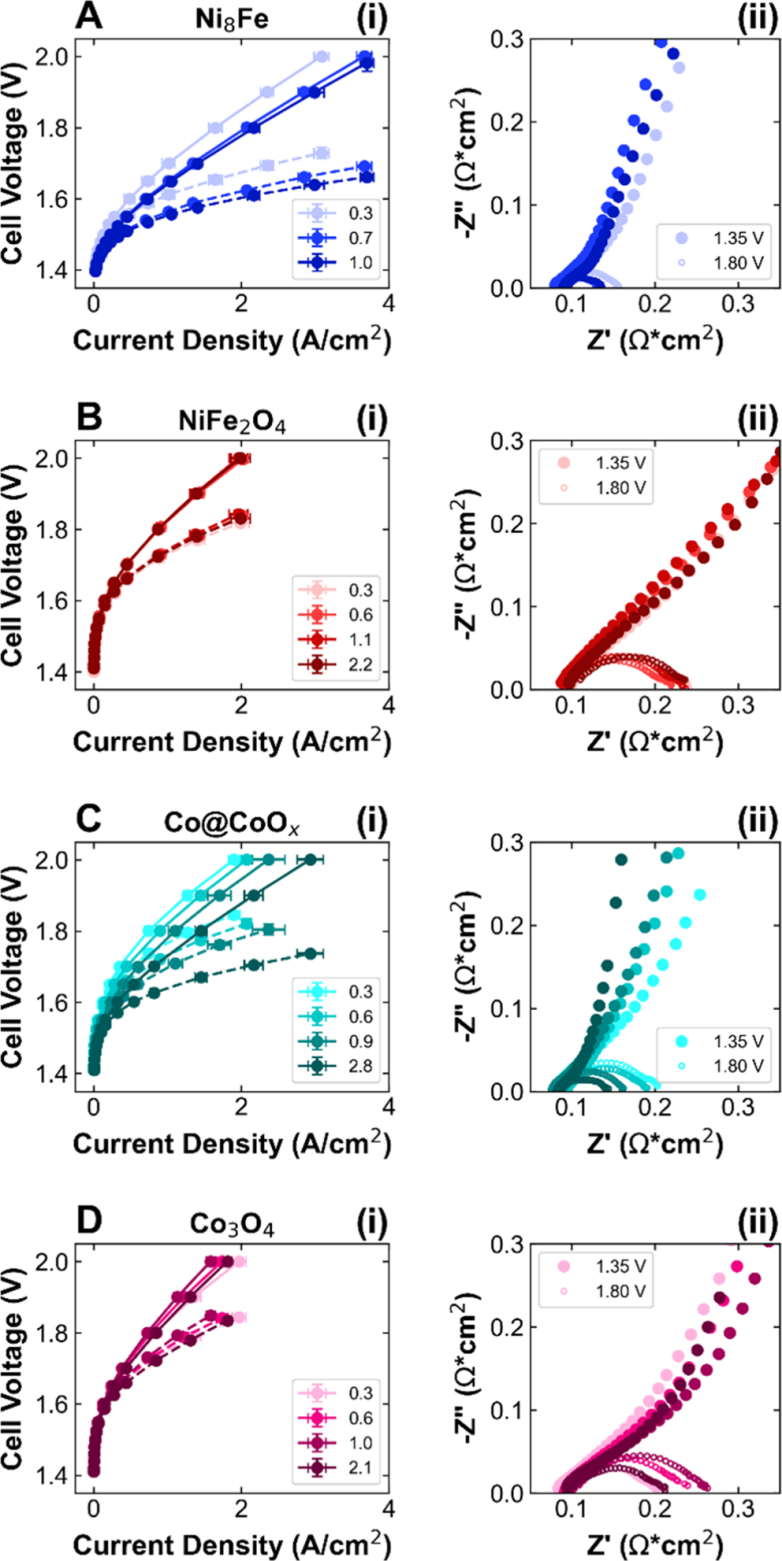
Performance of anode catalyst layers as a function of loading. (i) Polarization curves with (dashed lines) and without (solid lines) HFR-correction and (ii) Nyquist plot of electrochemical impedance spectra at 1.35 and 1.8 V (exact loadings in mg/cm^2^ given in labels) for (A) Ni_8_Fe (blue), (B) NiFe_2_O_4_ (red), (C) Co@CoO_*x*_ (teal), and (D) Co_3_O_4_ (pink). Performance data is reported in triplicate; EIS spectra are representative of the average behavior. Darker colors correspond to higher loadings.

For Ni_8_Fe, which has the most uniform and thickest catalyst layers, as well as the lowest sheet resistance, we observe an improvement in performance with increasing loading from 0.3 to 0.9 mg/cm^2^ (2 mg/cm^2^ loading was not possible due to problems with adhesion to the PTL). Figure 5A(i) shows a substantial increase in current density at 2 V from 3.09 to 3.87 A/cm^2^ and a decrease in the voltage at 1 A/cm^2^ from 1.609 to 1.553 V (HFR-free). As shown in the Nyquist plots (Figure 5A(ii)), there are minimal differences in HFR, resulting in overlaid ohmic losses (Figure S17) and identical performance trends for uncorrected and HFR-free data. At higher loadings, the EIS at 1.8 V shows a decrease in charge transfer resistance in agreement with the higher observed current densities, while the EIS at 1.35 V indicates a decrease in catalyst layer resistance (summarized in Table S1). In contrast, NiFe_2_O_4_ shows almost no change in performance as a function of loading (Figure 5B(i)). The current density at 2 V is ∼2 A/cm^2^ for the four loadings, with variations between loadings smaller than the variation within the samples with the same loading. There is no trend of change in HFR (Figure 5B(ii)) or ohmic losses (Figure S18) with loading, resulting in overlapping HFR-free polarization curves.

Co@CoO_*x*_ shows similar behavior to Ni_8_Fe, with performance improving significantly as loading increases (Figure 5C(i)). From 0.3 to 2.8 mg/cm^2^ loading, the current density at 2 V increases from 1.90 to 2.94 A/cm^2^, and the HFR-free voltage at 1 A/cm^2^ decreases by ∼125 mV from 1.766 to 1.640 V. The Nyquist and ohmic loss plots (Figures 5C(ii) and S19) show insignificant HFR changes as a function of loading, indicating that through-plane resistance is not increasing substantially. However, the EIS spectra at 1.25 and 1.8 V show significant decreases in catalyst layer resistance and charge transfer resistance, respectively, as loading increases (Table S1). Finally, the relationship between loading and performance for Co_3_O_4_ is slightly more complicated. From 0.3 to 1.0 mg/cm^2^, overall performance declines (Figure 5D(i)), with current density at 2 V decreasing from 1.97 to 1.59 A/cm^2^ and the voltage at 1 A/cm^2^ increasing from 1.75 to 1.77 V (HFR-free). There are differences in HFR, with ∼80 mΩ cm^2^ for 0.3 mg/cm^2^ increasing to ∼90 mΩ cm^2^ for the higher loadings, with corresponding differences in ohmic loss (Figures 5D(ii) and S20). Interestingly, at the highest loading of 2.1 mg/cm^2^, the overall performance is similar to that at 0.3 mg/cm^2^, achieving 1.81 A/cm^2^ at 2 V and an HFR-free voltage of 1.74 V at 1 A/cm^2^.While the HFR remains higher at 91 mΩ cm^2^, there is a clear decrease in charge transfer resistance compared to the intermediate loadings. To better understand the origins of these performance trends, we will next consider the effect of loading on kinetics and catalyst layer resistances.

**Figure 6:**
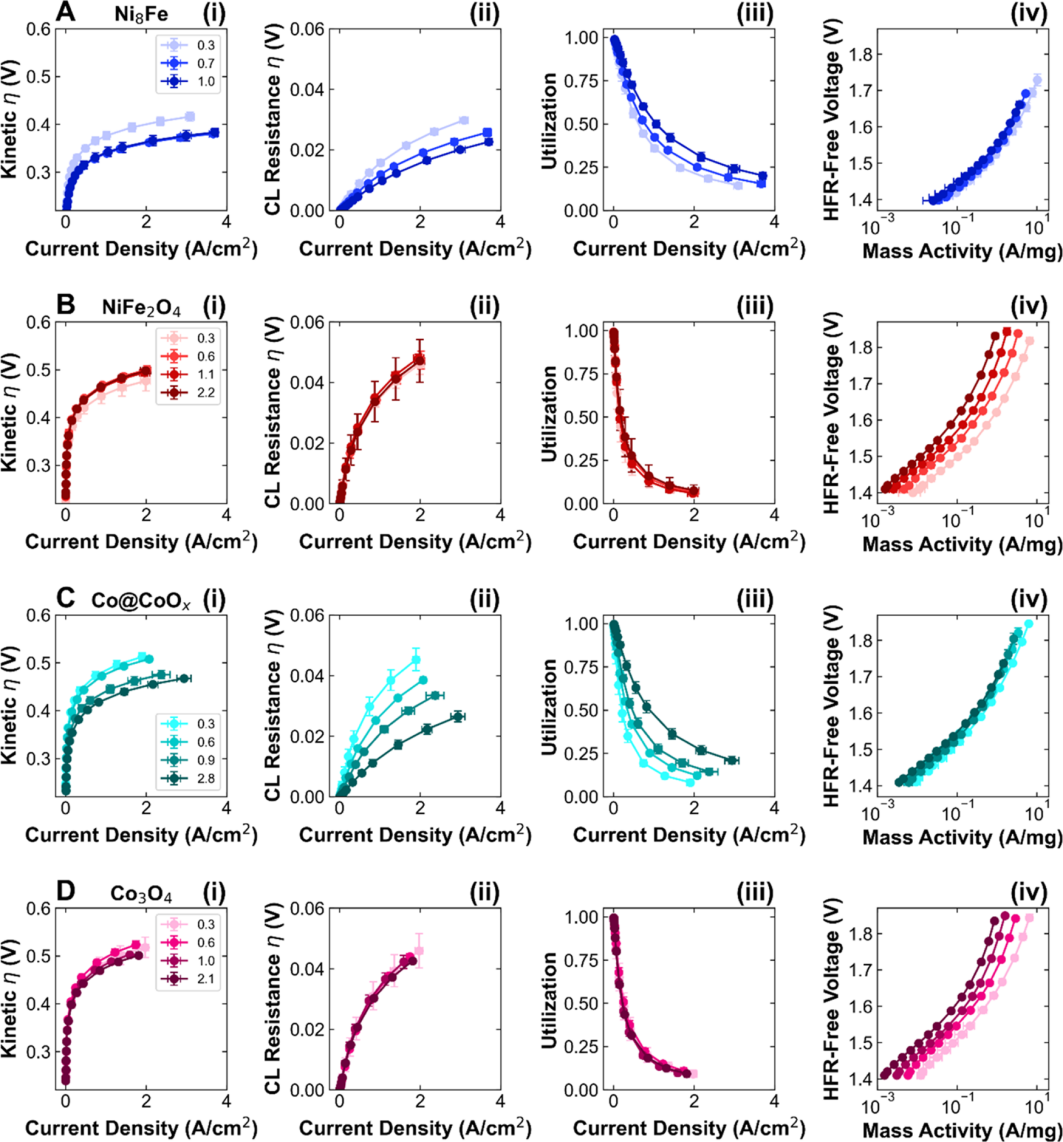
Kinetic and catalyst layer resistance effects as a function of loading. (i) Kinetic overpotential, (ii) catalyst layer resistance overpotential, (iii) catalyst utilization, and (iv) mass activity as a function of current density and anode catalyst loading from 0.3 to >2+ mg/cm^2^ for (A) Ni_8_Fe (blue), (B) NiFe_2_O_4_ (red), (C) Co@CoO_*x*_ (teal), and (D) Co_3_O_4_ (pink). Performance data is reported in triplicate. Darker colors correspond to higher loadings.

Figure 6 shows the kinetic losses, catalyst layer resistance losses, catalyst utilization, and mass activity for each catalyst. For Ni_8_Fe, the kinetic losses decrease sharply from 0.3 to 0.6 mg/cm^2^, corresponding to a decrease in *V*–log(*I*) slope from 82 to 73 mV/dec (Figure 6A(i)). As shown in Figure 5A(ii), the *R*_CL_ also decreases with increased loading from 150 to 80 mΩ cm^2^, resulting in reduced *R*_CL_ losses and increased catalyst utilization (Figure 6A(ii,iii)). This improvement in kinetics and catalyst layer utilization as catalyst layer thickness increases corresponds well to the high conductivity model shown in Figure 3A–C, wherein the improvement in in-plane electronic conductivity with a more cohesive catalyst layer results in more accessible active sites and an overall decrease in *R*_CL_. The mass activity is very similar across the three loadings, indicating that the extra catalyst loading is electrochemically accessible. In contrast, NiFe_2_O_4_ shows no improvement to the kinetics with loading (Figure 6B(i)). There is a slight increase in *R*_CL_ at higher loadings, but this has minimal effects on the catalyst layer resistance losses or utilization (Figure 6B(ii,iii)). Furthermore, Figure 6B(iv) shows that the mass activity decreases significantly with loading, indicating that the extra catalyst loading is not contributing to performance. This catalyst thus generally corresponds to the low conductivity model in Figure 3D–F, in which increases in through-plane resistance with higher catalyst layer thicknesses lead to higher *R*_CL_ and make the extra catalyst loading unusable for OER.

Matching the trend of Ni_8_Fe, Co@CoO_*x*_ shows improvements in both kinetics and catalyst layer resistance with increased loading (Figure 6C(i,ii)). Between 0.3 and 2.8 mg/cm^2^, the *V*–log(*I*) slope decreases from 95 to 90 mV/dec and the *V*–log(*I*) intercept increases from 6 to 11 μA/cm^2^. Similarly, the *R*_CL_ decreases nearly 5-fold from 520 to 110 mΩ cm^2^ (Figure 5C(ii)), resulting in increased catalyst utilization (Figure 6C(iii)). The low CL thickness and poor uniformity (Figure 4C(i)) at low loading support the hypothesis of performance being limited by few active sites and poor in-plane conductivity. At higher loadings, there is an increase in the number of accessible sites and a decrease in overall resistance through the catalyst layer. This catalyst therefore corresponds to the high conductivity model shown in Figure 3A–C, wherein we assume that the improvement in in-plane electronic conductivity outweighs any increase in the through-plane conductivity due to the increase in the catalyst layer thickness. Like Ni_8_Fe, the mass activity is constant across the 0.3–0.9 mg/cm^2^ loadings; however, it decreases slightly at 2.8 mg/cm^2^, indicating that there is likely a limitation to how much catalyst can effectively be used (Figure 6C(iv)). Co_3_O_4_, however, shows only small changes in the kinetic and *R*_CL_ losses, as well as catalyst utilization, as a function of loading (Figure 6D(i–iii)). Between 0.3 and 2.1 mg/cm^2^, the *V*–log(*I*) slope decreases from 99 to 95 mV/dec and the *V*–log(*I*) intercept decreases from 9 to 6 μA/cm^2^, small and counter-acting changes that result in similar kinetic losses. There is no trend or significant variation in *R*_CL_, remaining between 460 and 510 mΩ cm^2^ for all loadings (Figure 5D(ii)). Because the number of accessible active sites does not increase with increased loading, the mass activity decreases with loading (Figure 6D(iv)). Between 0.3 and 1 mg/cm^2^, this catalyst corresponds well with the low conductivity model proposed in Figure 3D–F, with performance decreasing due to increased HFR and transport losses (Figure S20) related to through-plane resistances. At 2.1 mg/cm^2^ loading, however, the performance is comparable to that of the lowest loading, due to decreased kinetic and *R*_CL_ losses. Cross-sectional images of this high loading (Figure S14) show that the catalyst layer has improved homogeneity and PTL coverage, which likely allows for better in-plane conductivity and active site accessibility. Thus, the morphology of the catalyst layer is closely related to the effective in-plane and through-plane conductivity, with significant effects on active site accessibility and performance.

### 3.4 Catalyst Layer Stability

Previous studies of OER catalysts and anode catalyst layers have shown substantial changes to catalyst structure and oxidation state, ionomer content, and catalyst (layer) morphology after testing.^18−20,38,40,61^ These catalyst layer changes are particularly relevant to this study, as we aim to relate performance to properties of the catalyst layer and therefore need to understand how the anode may change during testing. In short-term durability tests at 2 V (0.6 mg/cm^2^ loading, details of the tests given in the SI), the four anode catalysts have demonstrated different stability profiles. As shown in Figure S21, NiFe_2_O_4_ and Co_3_O_4_ exhibit slight improvements in performance after the 2 V hold, with a ∼3% increase in current density at 2 V, primarily due to decreases in residual losses. In contrast, Ni_8_Fe and Co@CoO_*x*_ show slight decreases in current density at 2 V of 7 and 4%, respectively, after the stability test. The voltage breakdown analysis reveals a few different changes, including increases in kinetic and residual losses and decreases in ohmic and *R*_CL_ losses. To understand how these performance changes may relate to changes to the anode catalyst particles and/or catalyst layer, the anodes were characterized after testing using XPS, XRD, TEM, and SEM.

**Figure 7:**
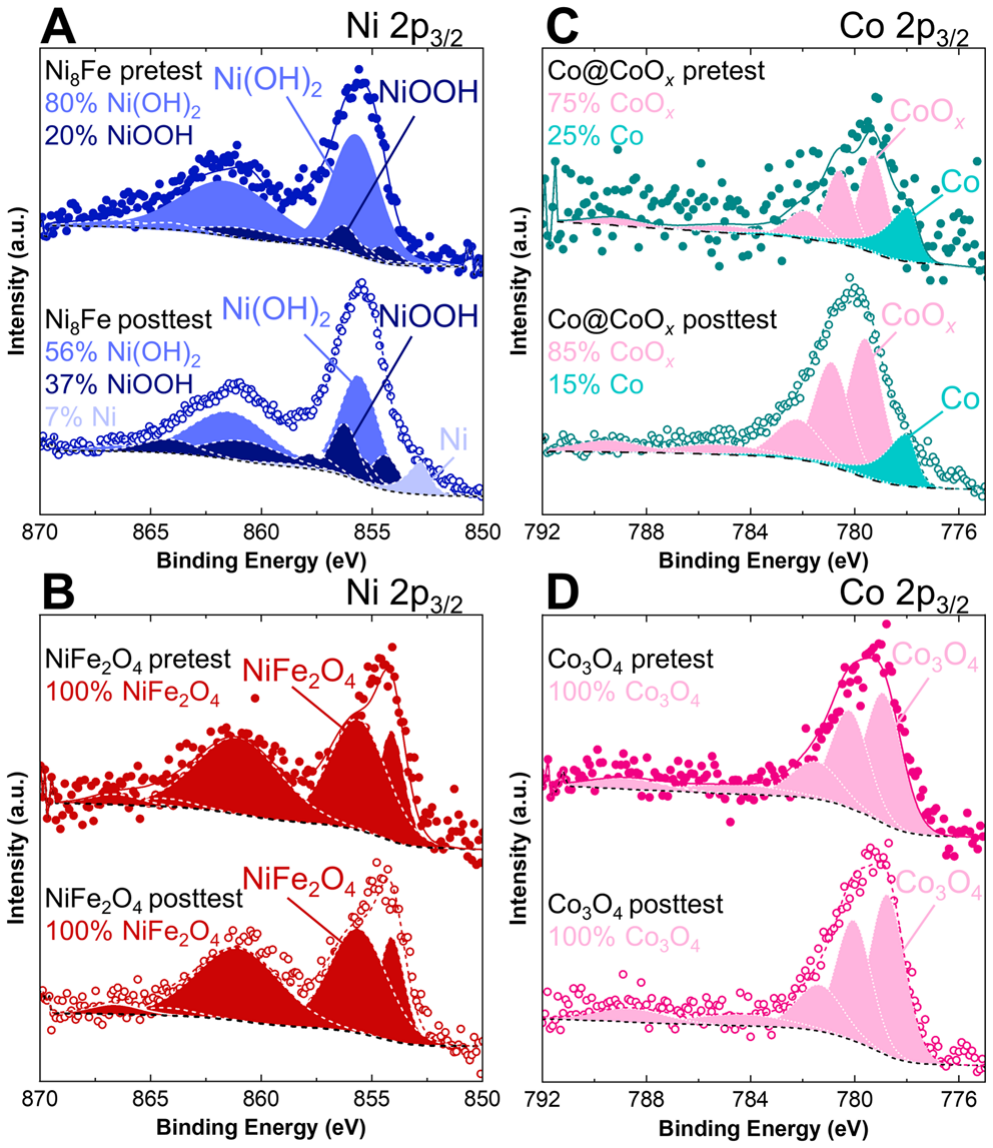
XPS characterization of anode catalysts after testing. Pre and posttest Ni 2p_3/2_ XPS spectra for (A) Ni_8_Fe (blue) and (B) NiFe_2_O_4_ (red), scraped off of the PTL to avoid background Ni signal, fits based on literature.^48^ Pre and posttest Co 2p_3/2_ XPS spectra for (C) Co@CoO_*x*_ (teal) and (D) Co_3_O_4_ (pink), measured directly on the PTL, fits based on literature.^48^ Test details: Ni_8_Fe = 1.0 mg/cm^2^ loading, constant current hold at 1 A/cm^2^ for 110 h; NiFe_2_O_4_ = 1.1 mg/cm^2^ loading, constant voltage hold at 2 V for 13 h; Co@CoO_*x*_ = 0.3 mg/cm^2^ loading, constant voltage hold at 2 V for 9 h; and Co_3_O_4_ = 0.3 mg/cm^2^ loading, constant voltage hold at 2 V for 18 h.

Focusing first on the catalyst particles, XPS was used to determine changes in oxidation state and composition, as well as ionomer degradation through the loss of N and F content. Figure 7A shows pre and posttest Ni 2p_3/2_ spectra for Ni_8_Fe. Prior to testing, the sample is a mix of Ni(OH)_2_ and NiOOH, giving an average oxidation state of Ni^2.2+^. After testing, the proportion of NiOOH increases, resulting in a modest increase to an oxidation state of Ni^2.4+^. It should be noted that these measurements are taken ex situ, with the catalyst removed from applied voltage and exposed to air, meaning that they may not capture the actual oxidation states that exist during operation. In both pre and posttest, the Fe 2p signal is too low to analyze. EDS analysis, however, indicates that the Ni-to-Fe ratio remains approximately constant at 7.5:1, with a slight decrease in the metal-to-O ratio. For NiFe_2_O_4_ (Figures 7B and S22), XPS shows no significant changes, with both the Ni 2p_3/2_ and Fe 2p_3/2_ spectra fitting well to NiFe_2_O_4_, giving average oxidation states of Ni^2+^ and Fe^3+^. The Ni-to-Fe ratio increases slightly from 1.2:1 to 1.5:1, indicating that further Ni surface enrichment occurs during testing, possibly due to Fe leaching from the nanoparticles. EDS confirms the change in Ni-to-Fe ratio, showing a substantially lower Fe content after testing but a constant metal-to-O ratio. This increase in the Ni-to-Fe ratio may contribute to the improved kinetics after the durability test (Figure S20), as lower Fe content than the stoichiometric 1:2 Ni-to-Fe ratio has been shown to be beneficial for OER activity in NiFe catalysts.^20^ Co@CoO_*x*_ had a mixed metallic Co and CoO_*x*_ composition prior to testing; the proportion of CoO_*x*_ increased slightly after testing (Figure 7C). In addition, after testing, significant Ni 2p peaks are visible posttest, indicating decreased PTL coverage, while F 1s and N 1s signal is drastically decreased, corresponding to ionomer loss and/or degradation (Figure S22). EDS shows a substantial decrease in the Co-to-O ratio from 4:1 before testing to 0.9:1 after testing. Finally, XPS shows that Co_3_O_4_ does not change with testing (Figure 7D), and EDS confirms that the Co-to-O ratio remains at 4:5.

**Figure 8:**
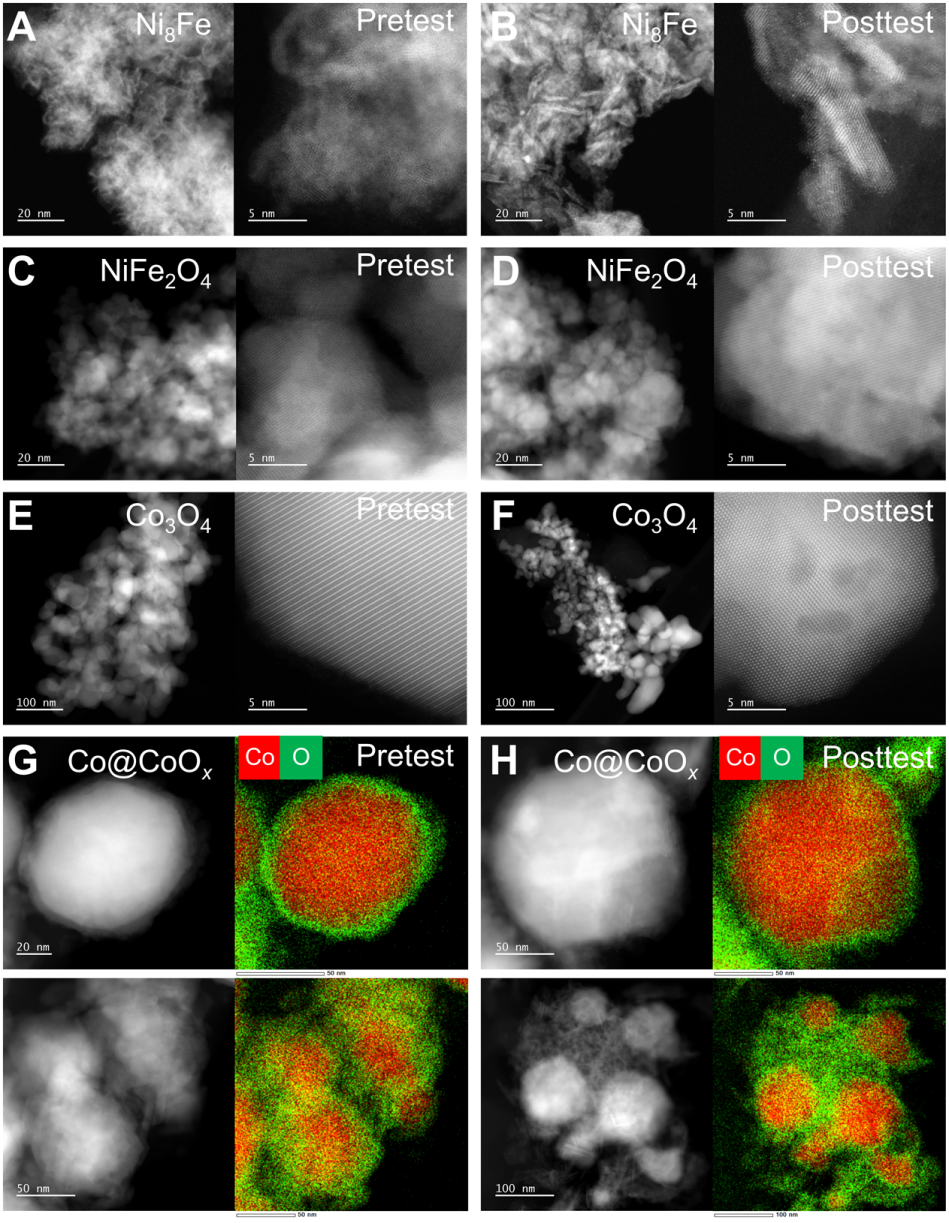
TEM characterization of anode catalysts after testing. Pre (A, C, E) and posttest (B, D, F) HAADF-STEM images for (A, B) Ni_8_Fe, (C, D) NiFe_2_O_4_, and (E, F) Co_3_O_4_. Pre (G) and posttest (H) HAADF-STEM images and EDS maps for Co@CoO_*x*_ showing representative particle morphologies and oxidized shell. Test details: Ni_8_Fe = 1 mg/cm^2^ loading, constant current hold at 1 A/cm^2^ for 110 h; NiFe_2_O_4_ = 1 mg/cm^2^ loading, constant voltage hold at 2 V for 12 h; Co@CoO_*x*_ = 0.6 mg/cm^2^ loading, constant voltage hold at 2 V for 30 h; and Co_3_O_4_ = 0.6 mg/cm^2^ loading, constant voltage hold at 2 V for 35 h.

HAADF images of the Ni_8_Fe particles (sonicated off of the PTL) show a change from small, randomly oriented crystallites within amorphous particles to needle-like structures and larger crystal grains within more sharply defined particles (Figures 8A,B and S23). Posttest XRD shows a small shift in peaks, which may reflect the formation of crystalline Ni(OH)_2_ (Figure S24). In contrast, NiFe_2_O_4_ shows minimal change in particle morphology and retains a high degree of crystallinity (Figures 8C,D, S24, and S25). Similarly, Co_3_O_4_ shows some particle coarsening and agglomeration, but there is minimal change to particle shape or crystallinity (Figures 8E,F and S26). Finally, TEM analysis for Co reveals a mix of spherical particles, which retain size and morphology similar to the pretest, needle-like structures, and larger agglomerates with varied shapes and sizes (Figures 8G,H and S27). Notably, the spherical particles retain the discernible metal core-oxide shell structure, while the other structures exhibit more complete oxidation. XRD shows that fcc Co is the only crystalline phase, indicating that the oxidized structures are amorphous (Figure S24). To summarize, the performance of the NiFe_2_O_4_ and Co_3_O_4_ anodes improves slightly over time, but other than an increase in Ni-to-Fe ratio for NiFe_2_O_4_, there are no significant catalyst particle changes. In contrast, Ni_8_Fe and Co@CoO_*x*_ show slight performance degradation and undergo material changes, notably oxidation and morphology changes, including the formation of similar needle-like structures.

To understand these changes in the context of the full anode, characterization of the full catalyst layer is needed. It is important to note that compression within the cell and the process of disassembly can affect the catalyst layer morphology, such as by transferring some of the catalyst layers to the membrane, so observed changes between the catalyst layers before and after testing should not be fully attributed to the effects of testing. However, the anodes were all handled in the same manner, so comparison is still valuable. Cross-sectional and top-down SEM images of the Ni_8_Fe anode posttest show a thinner catalyst layer, with less coverage of the PTL fibers (Figure S28). The Co@CoO_*x*_ catalyst layer is also thinner posttest, but it appears to have increased in density while maintaining similar PTL coverage (Figure S29). This apparent loss of material and densification for these two anodes may relate to the observed increases in kinetic and residual overpotential (Figure S21), such as through the loss of active sites and decreased porosity that leads to worsened mass transport. It is possible that the substantial particle-level morphological changes contribute to the observed thinning and densification of the catalyst layers. In contrast, the Co_3_O_4_ and NiFe_2_O_4_ particles were more stable, and their catalyst layers also showed less change. Co_3_O_4_ has large particle agglomerates and a heterogeneous catalyst layer, both pre and posttest (Figure S30). Finally, NiFe_2_O_4_ does not show significant changes to catalyst layer thickness or morphology (Figure S31), indicating that the performance enhancement over time is likely due to an increase in intrinsic activity and/or electrochemically accessible surface area as Fe is lost from the catalyst. Although it is difficult to conclusively assign differences in performance to certain catalyst or catalyst layer properties, this analysis shows that characterization is vital to understanding the distinct behaviors of these catalysts.

## 4 Conclusions

Improvements to anion exchange polymers and PGM-free catalysts have helped to bridge the gap between AEMWE and the more established low-temperature electrolysis technologies. The catalyst layer architecture offers important advantages over the electrodes employed for traditional alkaline electrolysis, particularly the improved active site accessibility due to nanostructured catalysts and high material density near the membrane. However, an improved understanding of the impact of material properties and integration strategies on cell efficiency and durability is needed to capitalize on these advantages and reach performance targets. Through the investigation of several anode catalysts, we have demonstrated that different catalysts form catalyst layers with distinct material properties. Voltage breakdown analysis indicated that the difference in performance between catalysts reflects both intrinsic OER kinetics and resistances within the catalyst layer that determine catalyst utilization. Catalyst loading was proposed as an important variable to tune performance by providing additional catalyst sites and decreasing the in-plane resistance that results from large fiber-to-fiber distances in the porous transport layer and gaps in the catalyst layer. Top-down and cross-sectional microscopy showed that catalyst layer thickness, coverage, and uniformity increased with increasing loading for all catalysts. For the catalysts with high electronic conductivity, Ni_8_Fe, and Co@CoO_*x*_, this increase in loading resulted in improved kinetics, catalyst layer resistance, and utilization, leading to significant performance improvements with loading. For the less conductive catalysts, NiFe_2_O_4_ and Co_3_O_4_, however, increased loading had minimal impact on performance. In short-term durability testing, Ni_8_Fe and Co@CoO_*x*_ showed slight decreases in performance primarily due to worsened kinetics, while the catalyst particles increased in surface oxidation and underwent morphological changes. In contrast, NiFe_2_O_4_ and Co_3_O_4_ improved slightly in performance due to improvements in kinetics and exhibited minimal changes to the catalyst particles or catalyst layer structure. These results indicate a possible trade-off between activity and durability related to initial catalyst material properties that should be studied further. This work shows that AEMWE catalyst layer design should be informed, at minimum, by the catalyst conductivity, catalyst layer uniformity and coverage, and porous transport layer morphology.

## Supporting Information Available

Additional experimental details and calculations, catalyst characterization, and electrochemical measurements (PDF)
