# Supplementary material for: Understanding the Effects of Anode Catalyst Conductivity and Loading on Catalyst Layer Utilization and Performance for Anion Exchange Membrane Water Electrolysis
Source: ACS Catal. 2024 Jul 3;14(14):10806–19. doi: 10.1021/acscatal.4c02932 (PMC11264204; doi:10.1021/acscatal.4c02932)
Supplement: Supplementary file 1 — cs4c02932_si_001.pdf [file cs4c02932_si_001.pdf]

Supporting Information

Understanding the Effects of Anode Catalyst Conductivity and Loading on Catalyst Layer Utilization and Performance for Anion Exchange Membrane Water Electrolysis

Melissa E. Kreider[1], Haoran Yu[2], Luigi Osmieri[3], Makenzie R. Parimuha[1], Kimberly S. Reeves[2], Daniela H. Marin[4,5], Ryan T. Hannagan[4,5], Emily K. Volk[6], Thomas F. Jaramillo[4,5], James L. Young[1], Piotr Zelenay[3], Shaun M. Alia[1,*]

[1] Chemistry and Nanoscience Center, National Renewable Energy Laboratory, Golden, CO 80401, United States

[2] Center for Nanophase Materials Sciences, Oak Ridge National Laboratory, Oak Ridge, TN 37830, United States

[3] Materials Physics and Applications Division, Los Alamos National Laboratory, Los Alamos, NM 87545, United States

[4] Department of Chemical Engineering, Stanford University, Stanford, CA 94305, United States

[5] SUNCAT Center for Interface Science and Catalysis, SLAC National Accelerator Laboratory, Menlo Park, CA 94025, United States

[6] Advanced Energy Systems Graduate Program, Colorado School of Mines, Golden, CO 80401, United States

* Corresponding author: shaun.alia@nrel.gov

Table of Contents

(1) Calculations

- Equations for Voltage Breakdown Analysis
- Equations for In-Plane Conductivity Calculation

(2) Tables

- **Table S1.** Summary of Anode Catalyst Layers and Performance

(3) Figures

- **Figure S1.** Pretest XRD of Ni_8Fe and $NiFe_2O_4$
- **Figure S2.** TEM and EDS maps of Ni_8Fe and $NiFe_2O_4$
- **Figure S3.** XPS spectra of Ni_8Fe and $NiFe_2O_4$
- **Figure S4.** In-plane conductivity measurements
- **Figure S5.** Voltage loss breakdown analysis for Ni_8Fe and $NiFe_2O_4$
- **Figure S6.** Cyclic voltammograms for Ni_8Fe and $NiFe_2O_4$
- **Figure S7.** Calculated R_{CL} values by two methods
- **Figure S8.** Pretest XRD of Co@CoO_x and Co_3O_4
- **Figure S9.** TEM of Co@CoO_x and Co_3O_4
- **Figure S10.** EDS maps of Co@CoO_x and Co_3O_4
- **Figure S11.** XPS spectra of Co@CoO_x and Co_3O_4
- **Figure S12.** Voltage loss breakdown analysis for Co@CoO_x and Co_3O_4
- **Figure S13.** Cyclic voltammograms for Co@CoO_x and Co_3O_4
- **Figure S14.** Cross-section SEM of catalyst layers at all loadings
- **Figure S15.** Cross-section EDS map of Ni_8Fe catalyst layer
- **Figure S16.** Cyclic voltammograms at various loadings
- **Figure S17.** Voltage loss breakdown analysis for Ni_8Fe at various loadings
- **Figure S18.** Voltage loss breakdown analysis for $NiFe_2O_4$ at various loadings
- **Figure S19.** Voltage loss breakdown analysis for Co@CoO_x at various loadings
- **Figure S20.** Voltage loss breakdown analysis for Co_3O_4 at various loadings
- **Figure S21.** AEMWE performance before and after durability tests
- **Figure S22.** XPS spectra after AEMWE testing
- **Figure S23.** TEM and EDS maps for Ni_8Fe after AEMWE testing
- **Figure S24.** Pre- and post-test XRD
- **Figure S25.** TEM and EDS maps for $NiFe_2O_4$ after AEMWE testing
- **Figure S26.** TEM and EDS maps for Co_3O_4 after AEMWE testing
- **Figure S27.** TEM and EDS maps for Co@CoO_x after AEMWE testing
- **Figure S28.** Pre and post-test SEM images of Ni_8Fe catalyst layers
- **Figure S29.** Pre and post-test SEM images of Co@CoO_x catalyst layers
- **Figure S30.** Pre and post-test SEM images of Co_3O_4 catalyst layers
- **Figure S31.** Pre and post-test SEM images of $NiFe_2O_4$ catalyst layers

Voltage Breakdown Analysis

The total overpotential can be described as:

$$\eta_{total} = \eta_{ohmic} + \eta_{kinetic} + \eta_{CLR} + \eta_{residual}$$

Ohmic overpotential describes the voltage loss due to ionic resistance through the membrane and contact resistances, or electronic resistance at the interfaces between the membrane, CLs, and PTL/GDL. Experimentally, this is measured as the high frequency resistance (HFR) in electrochemical impedance spectra (EIS). The overpotential is given by Ohm's law with total cell current:

$$\eta_{ohmic}[V] = J\left[\frac{A}{cm^2}\right] * HFR\ [\Omega * cm^2]$$

Next, the kinetic loss is due to the HER and OER overpotentials of the catalysts, which can be described using the Tafel equation, where b = slope of V-log(I) linear fit, Jo = intercept of V-log(I) linear fit, and E_{rev} is the reversible potential:

$$\eta_{kinetic}[V] = b\left[\frac{V}{dec}\right] * \log\left(\frac{I}{I_o}\right) - E_{rev}[V]$$

The equilibrium potential at the operating pressure (82 kPa) and temperature (80 °C) in this work is calculated using the Nernst equation[1,2]:

$$\begin{aligned} E_{rev}[V] &= E^o - \frac{\Delta S}{nF} * (T - T^o) - \frac{RT}{nF}\ln(Q) \\ &= 1.229\ [V] - \frac{\left(2 * S_{H_2} + S_{O_2} - 2 * S_{H_2O}\right)\left[\frac{J}{mol * K}\right]}{4\ mol\ e^- * 96485 \frac{C}{mol\ e^-}} * (80 + 273.15 - 298.15)[K] \\ &+ \frac{8.314\left[\frac{J}{mol * K}\right] * (80 + 273.15)\ [K]}{4\ [mol\ e^-] * 96485\left[\frac{C}{mol\ e^-}\right]} \ln\left(\frac{P_{H_2}^2 * P_{O_2}}{act_{H_2O} P_o^3}\right) \\ &= 1.229\ V - 0.00085 \frac{V}{K} * 55K + 0.0076V * \ln\left(\frac{82.2^3}{1 * 101.325^3}\right) = 1.178\ V \end{aligned}$$

The catalyst layer resistance (R_{CL}), which describes resistance to through-plane ionic and in- and through-plane electronic transport in the electrode, can be calculated by fitting EIS at non-Faradaic voltages, using a transmission line model. The main effect of this resistance is to decrease the catalyst layer utilization, which is described by the following equation[3]:

$$U\,(J) = \left(1 + \left(\frac{J\left[\frac{A}{cm^2}\right] * \ln(10) * R_{CL}[\Omega * cm^2]}{2b\left[\frac{V}{dec}\right]}\right)^{1.1982}\right)^{-1/1.1982}$$

The form of the equation indicates that catalyst layer utilization is lower at high current density, high values of R_{CL}, and low V-log(I) slopes. The overpotential due to this resistance is given by:

$$\eta_{CLR}[V] = -b\left[\frac{V}{dec}\right] * \log\big(U(J)\big)$$

Finally, the residual overpotential, which can be attributed to mass transport of water and product gases, is calculated as:

$$\eta_{residual}[V] = E_{cell} - E_{rev} - \eta_{ohmic} - \eta_{kinetic} - \eta_{CLR}$$

In-Plane Conductivity

Long gold ribbons with dimensions 18.5 x 0.25 mm were used to measure resistance in a system analogous to the classic 4-point probe. This method reduces the total flux of current between probe contacts and enables measurement of highly resistive systems. In the case of long ribbons (length >> width), current flux can be modelled as rectangular, and a correction factor was numerically derived, yielding a solution specific to the ribbon length and the equal spacing between the ribbons:

$$R_{sheet} = 12.8\frac{V}{I}$$

The percentage error was determined based on the non-idealities of the custom probe with inter-ribbon spacings of 0.8 ± 0.25 mm. An average of 30 measurements of each inter-ribbon spacing was used to inform a Monte Carlo simulation and determine the error associated with the standard deviation of the inter-ribbon spacing.

Table S1. Summary of Anode Catalyst Layers and Performance

Catalyst	Loading (mg/cm^2)	Thickness (μm)	V @ 1 A/cm^2 (HFR-free V)	HFR ($m\Omega*cm^2$)	R_{CL} ($m\Omega*cm^2$)
Ni_8Fe	0.32 ± 0.02	7.1 ± 2.7	1.609 ± 0.017	87 ± 3	146 ± 11
	0.66 ± 0.03	10.3 ± 3.2	1.564 ± 0.001	84 ± 2	103 ± 6
	0.96 ± 0.03	21.9 ± 6.2	1.553 ± 0.005	88 ± 4	77 ± 4
$NiFe_2O_4$	0.27 ± 0.03	1.8 ± 0.7	1.732 ± 0.014	89 ± 6	598 ± 8
	0.55 ± 0.07	4.5 ± 1.3	1.739 ± 0.001	82 ± 3	579 ± 52
	1.07 ± 0.00	5.8 ± 1.8	1.743 ± 0.007	84 ± 4	659 ± 29
	2.24 ± 0.03	14.5 ± 4.8	1.738 ± 0.005	88 ± 5	621 ± 244
$Co@CoO_x$	0.34 ± 0.00	1.4 ± 0.5	1.766 ± 0.009	83 ± 3	519 ± 83
	0.62 ± 0.04	2.8 ± 0.8	1.732 ± 0.010	87 ± 4	317 ± 28
	0.89 ± 0.01	6.6 ± 1.9	1.698 ± 0.017	81 ± 2	215 ± 3
	2.75 ± 0.09	10.9 ± 2.2	1.640 ± 0.004	89 ± 4	111 ± 13
Co_3O_4	0.31 ± 0.07	4.3 ± 1.3	1.752 ± 0.018	80 ± 1	481 ± 121
	0.60 ± 0.02	6.3 ± 1.8	1.758 ± 0.007	90 ± 4	455 ± 6
	0.95 ± 0.01	8.7 ± 3.7	1.766 ± 0.010	91 ± 6	458 ± 40
	2.07 ± 0.01	13.6 ± 3.8	1.741 ± 0.005	91 ± 2	468 ± 13

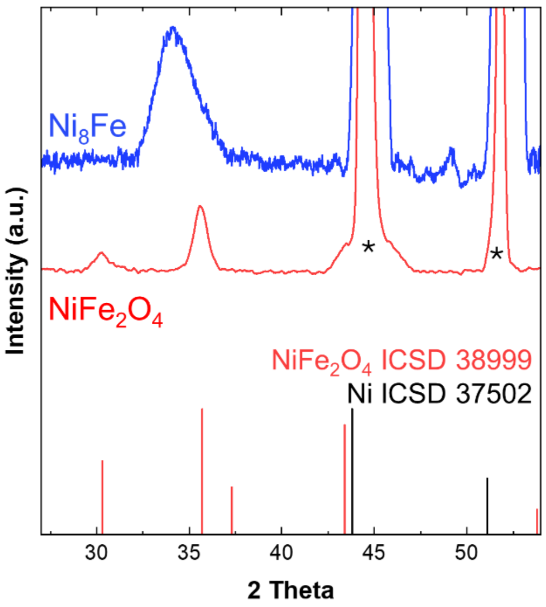

Figure S1. (**A**) Pretest XRD of Ni_8Fe (blue) and $NiFe_2O_4$ (red) sprayed on Ni PTLs. Reference patterns for $NiFe_2O_4$ (red sticks, ICSD 38999) and Ni (black sticks, ICSD 37502). Peaks from the Ni PTL are denoted with *.

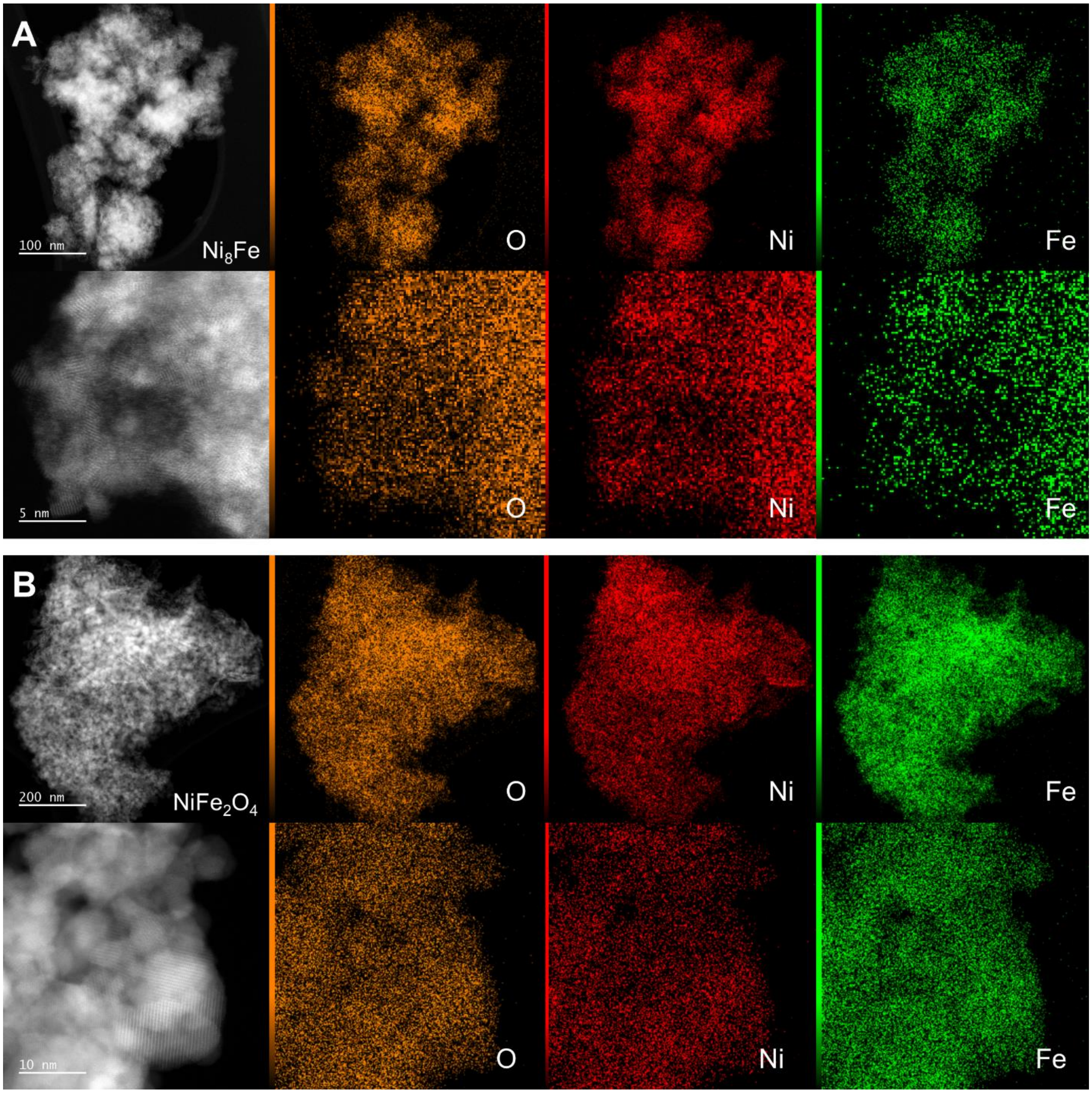

Figure S2. HAADF-STEM images and O, Ni, and Fe EDS maps for (**A**) Ni_8Fe and (**B**) $NiFe_2O_4$ particles, removed from the Ni PTL by sonication.

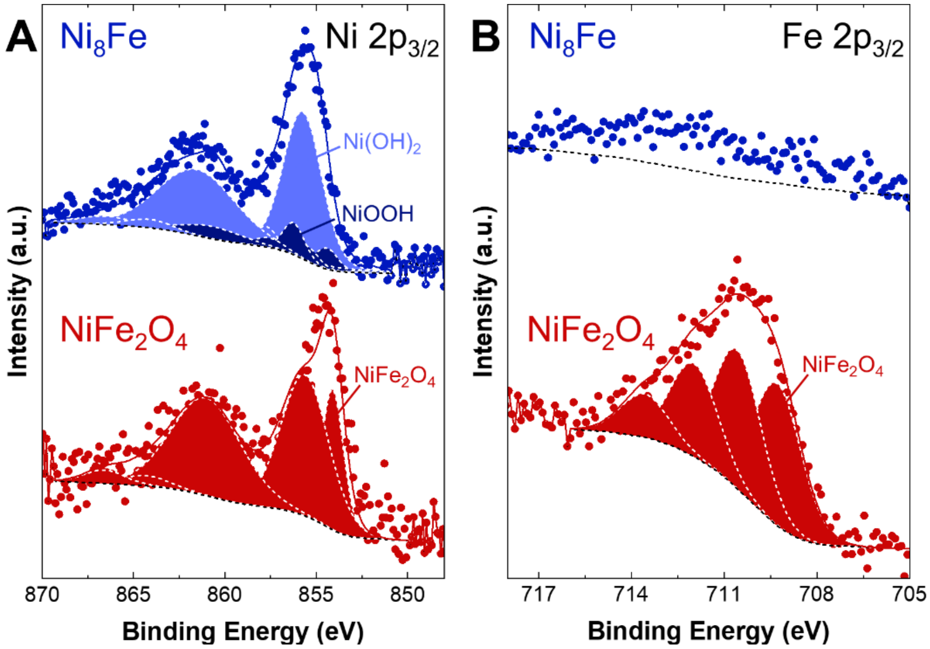

Figure S3. (**A**) Ni 2p and (**B**) Fe 2p XPS spectra of Ni_8Fe (blue) and $NiFe_2O_4$ (red) catalysts before testing, fits based on literature.[4]

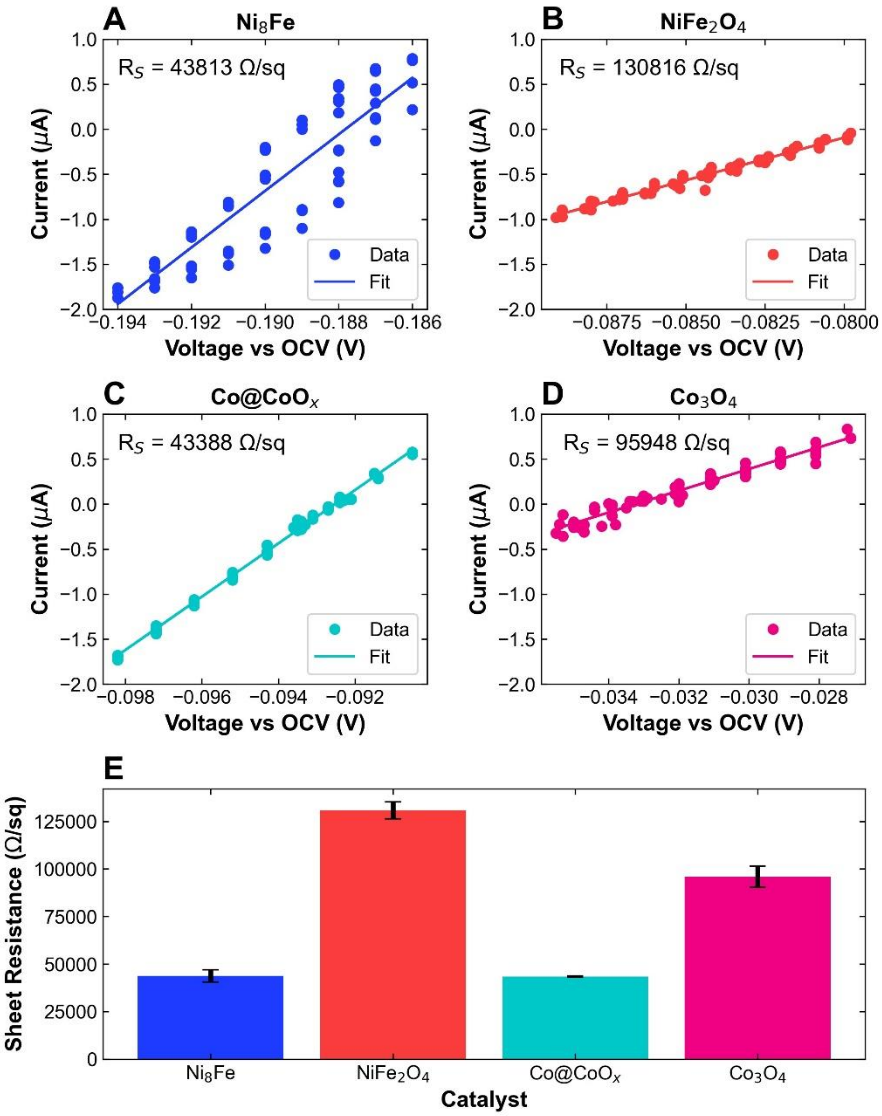

Figure S4. 4-point probe measurements of in-plane conductivity. Plots of current versus voltage for one cycle of the 4-point probe measurement with linear fit and calculated sheet resistance for (**A**) Ni_8Fe, (**B**) $NiFe_2O_4$, (**C**) Co@CoO_x, and (**D**) Co_3O_4. (**E**) Bar plot of calculated sheet resistance; average of 5 cycles.

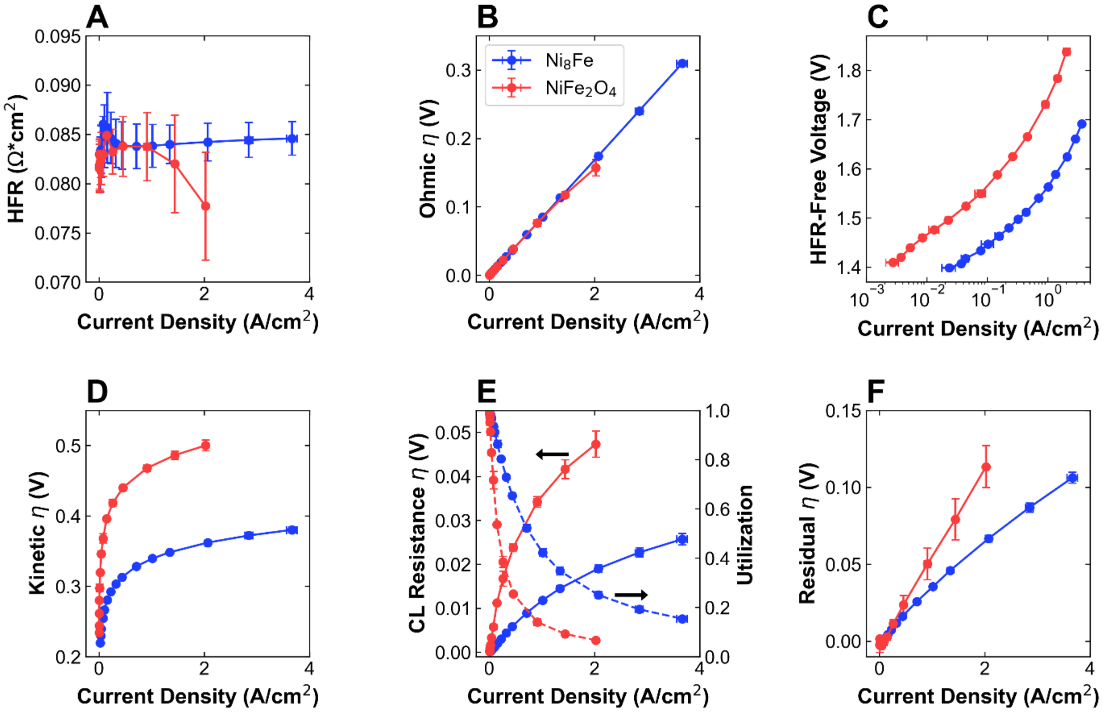

Figure S5. Voltage loss breakdown analysis for Ni_8Fe (blue) and $NiFe_2O_4$ (red) at ~0.6 mg/cm^2 loading, from **Figure 1**. As a function of current density: (**A**) HFR calculated from EIS, (**B**) ohmic overpotential, (**C**) HFR-free voltage-logarithm (current density) plot, (**D**) kinetic overpotential, (**E**) catalyst layer resistance overpotential (left, solid line) and utilization (right, dashed line), and (**F**) residual overpotential. Data is reported in triplicate.

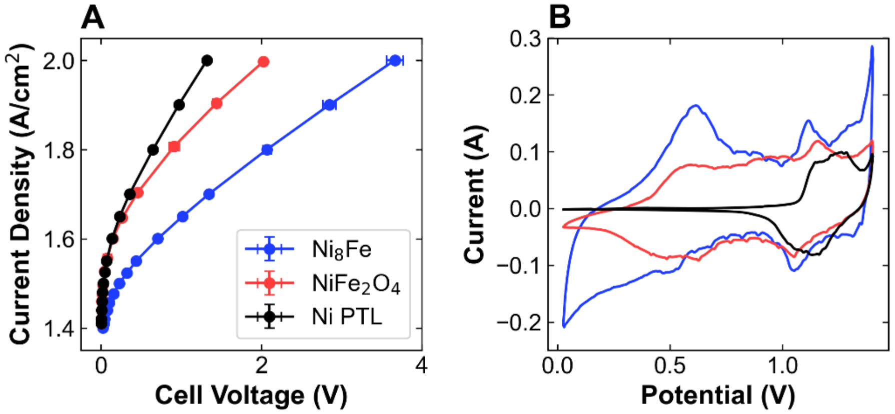

Figure S6. (**A**) Polarization curves and (**B**) cyclic voltammograms at 100 mV/s for Ni_8Fe (blue) and $NiFe_2O_4$ (red) anodes at ~0.6 mg/cm^2 loading, as well as an anode consisting of bare Ni PTL (black).

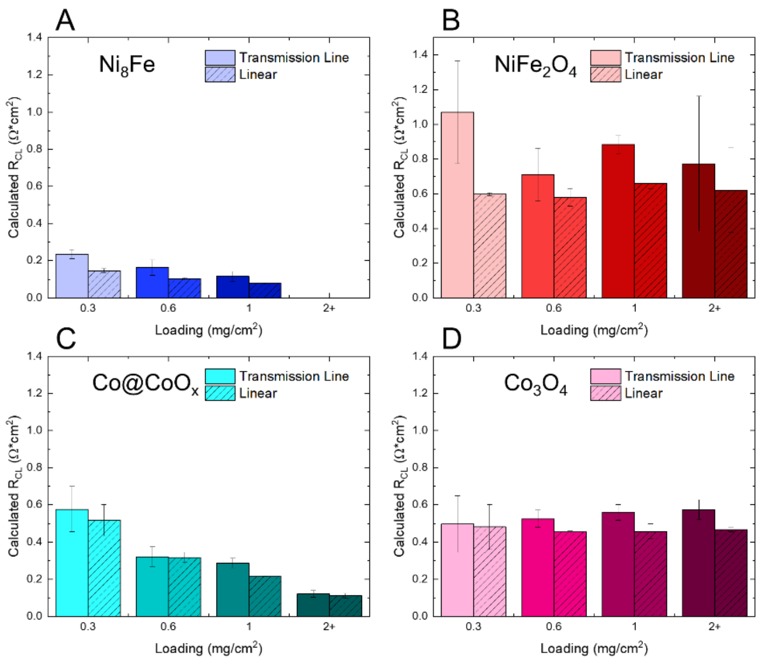

Figure S7. Calculated R_{CL} values using a transmission line curve fit (solid bar) and a linear intercept fit (dashed bar) for (**A**) Ni_8Fe, (**B**) $NiFe_2O_4$, (**C**) Co@CoO$_x$, and (**D**) Co_3O_4 at various loadings. Data is reported in triplicate.

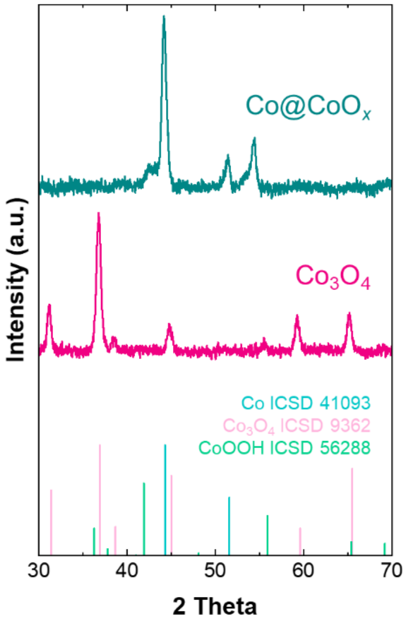

Figure S8. XRD of Co@CoO$_x$ (teal) and Co_3O_4 (pink) powders. Reference patterns for fcc Co (teal sticks, ICSD 41093), spinel Co_3O_4 (pink sticks, ICSD 9362), and CoOOH (green sticks, ICSD 56288).

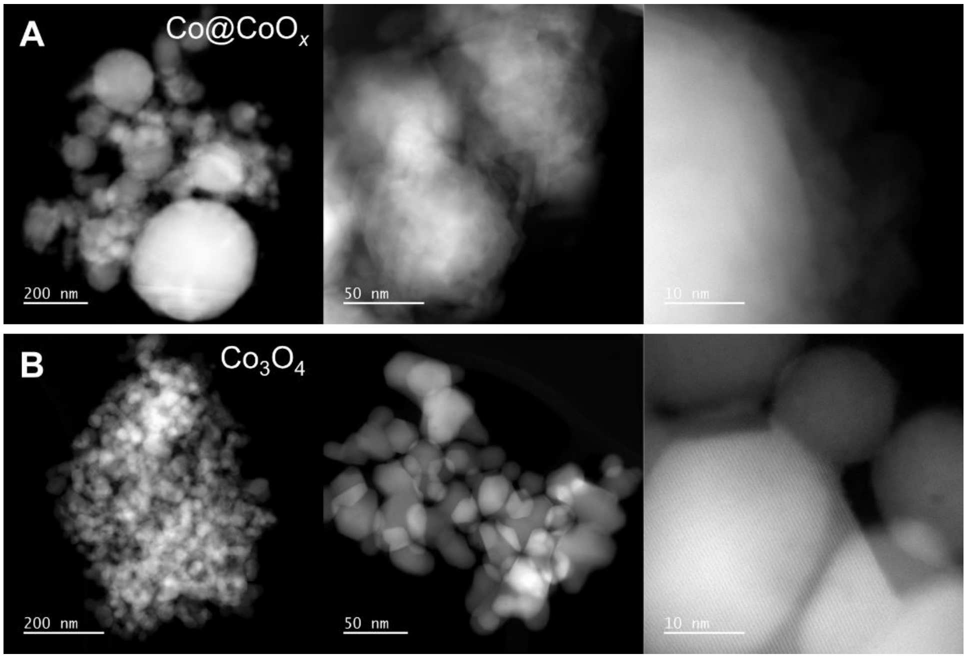

Figure S9. HAADF-STEM images for (**A**) Co@CoO$_x$ and (**B**) Co_3O_4 particles, removed from the Ni PTL by sonication. Co_3O_4 consists of smaller, more crystalline particles with defined edges, while Co@CoO$_x$ has a mix of particle shape and size and a core-shell structure.

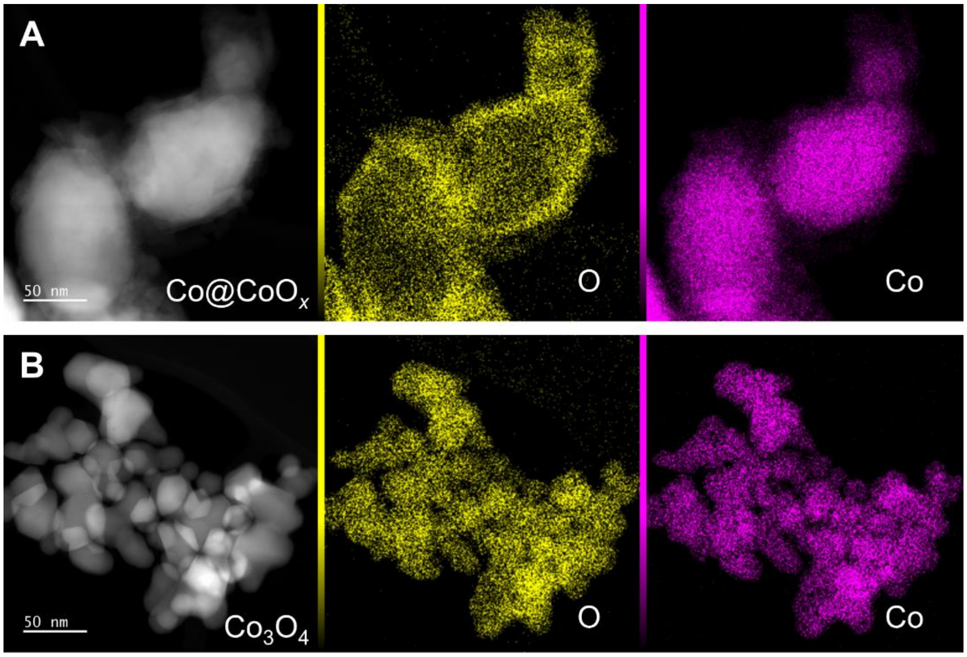

Figure S10. HAADF-STEM images and O and Co EDS maps for (**A**) Co@CoO$_x$ and (**B**) Co_3O_4 particles, removed from the Ni PTL by sonication. Co@CoO$_x$ shows larger particles with a metallic core and oxidized shell, while Co_3O_4 has agglomerates of smaller, fully oxidized particles.

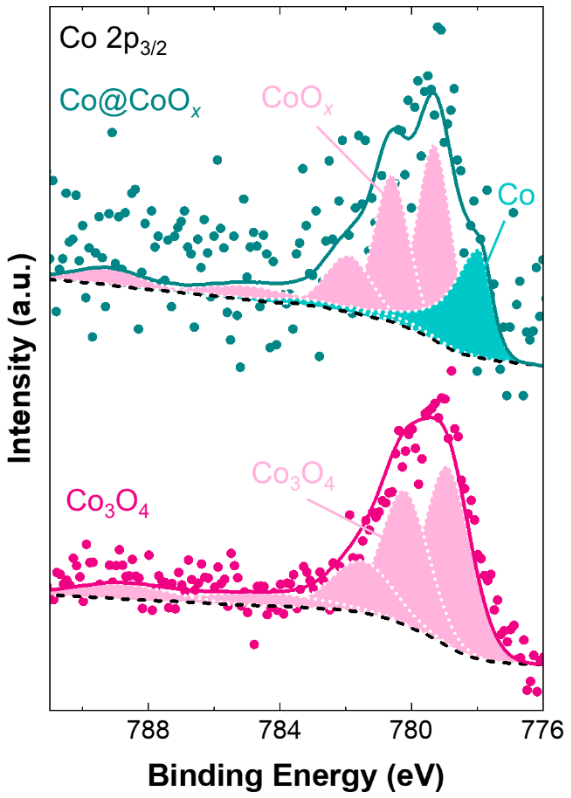

Figure S11. Co 2p XPS spectra of Co@CoO$_x$ (teal) and Co_3O_4 (pink) catalysts before testing, fits based on literature.[4]

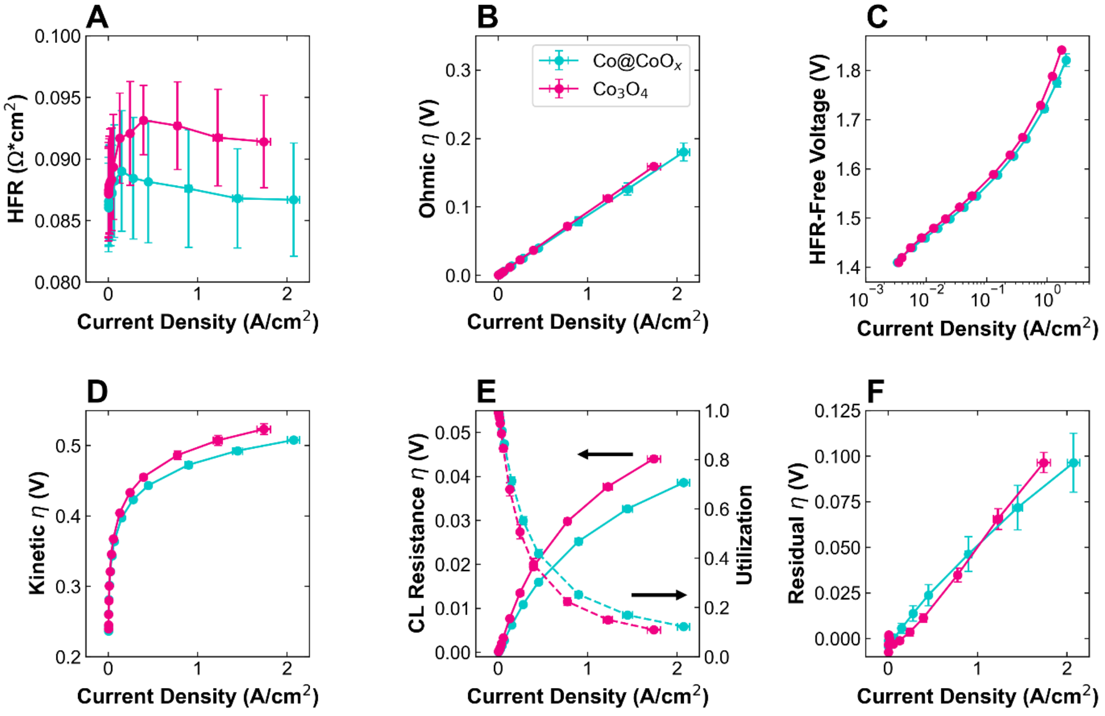

Figure S12. Voltage loss breakdown analysis for Co@CoO$_x$ (teal) and Co_3O_4 (pink) at ~0.6 mg/cm^2 loading, from **Figure 2**. As a function of current density: (**A**) HFR calculated from EIS, (**B**) ohmic overpotential, (**C**) HFR-free voltage-logarithm (current density) plot, (**D**) kinetic overpotential, (**E**) catalyst layer resistance overpotential (left, solid line) and utilization (right, dashed line), and (**F**) residual overpotential. Data is reported in triplicate.

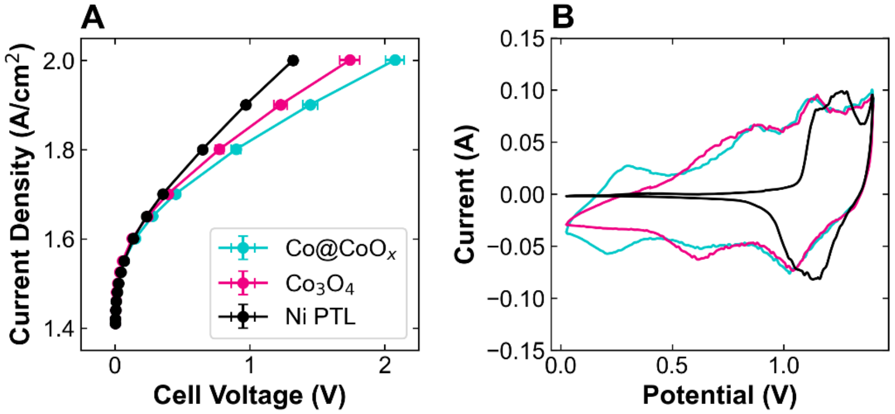

Figure S13. (**A**) Polarization curves and (**B**) cyclic voltammograms at 100 mV/s scan rate for Co@CoO$_x$ (teal) and Co_3O_4 (pink) anodes at ~0.6 mg/cm^2 loading, as well as an anode consisting of bare Ni PTL (black).

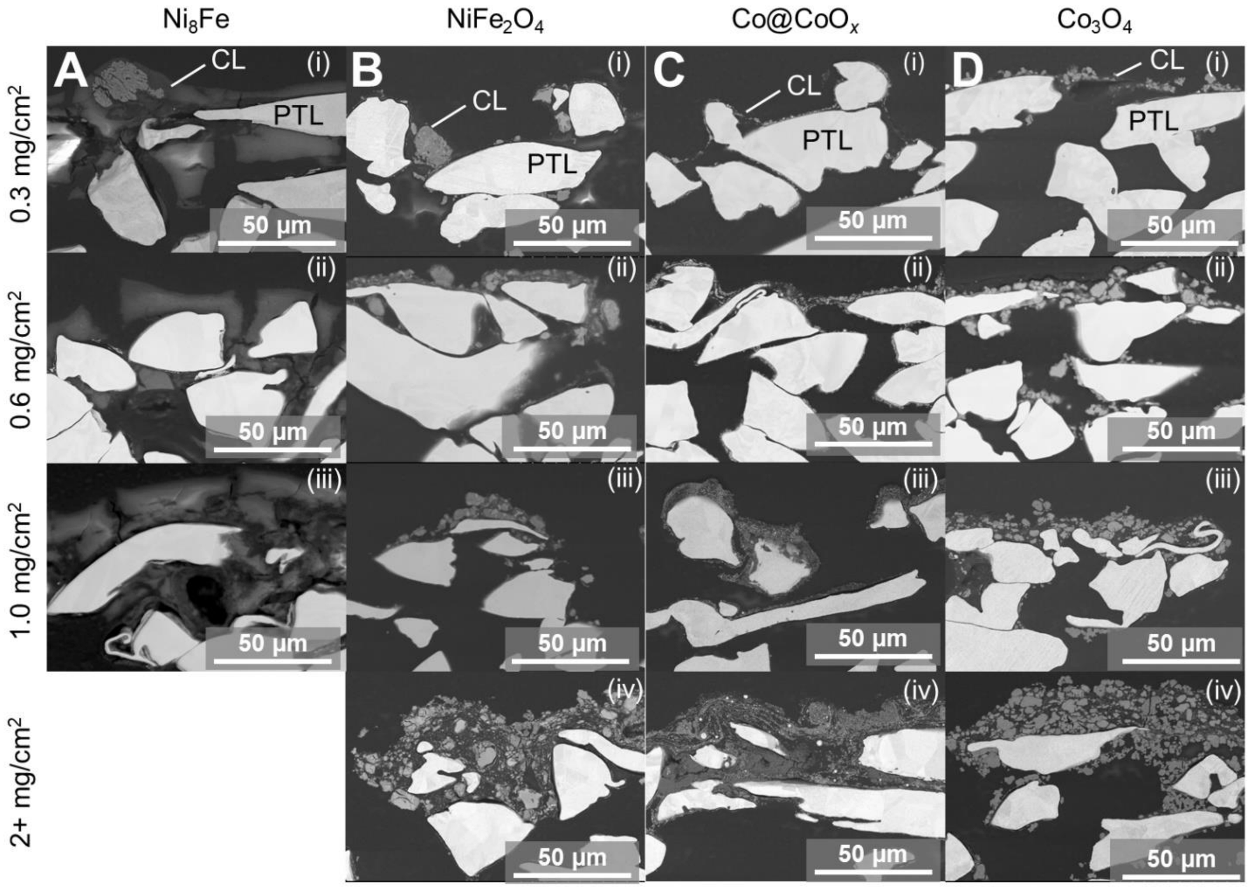

Figure S14. Cross-section SEM images of (**A**) Ni_8Fe, (**B**) $NiFe_2O_4$, (**C**) Co@CoO_x, and (**D**) Co_3O_4 anode catalyst layers at loadings of (i) 0.3 mg/cm^2, (ii) 0.6 mg/cm^2, (iii) 1 mg/cm^2, and (iv) 2+ mg/cm^2.

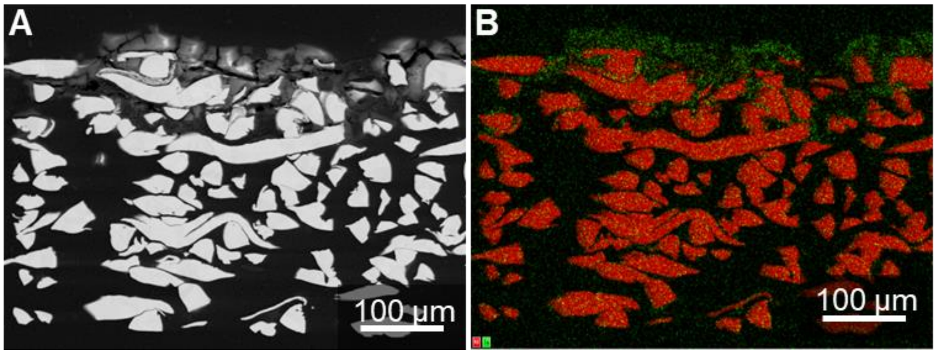

Figure S15. Cross-section (**A**) SEM image and (**B**) overlaid EDS Ni (red) and Fe (green) maps of 1 mg/cm^2 Ni_8Fe anode on Ni PTL. Because the PTL is Ni, Fe signal can be used to show the distribution of catalyst into the PTL, which is largely confined to the top third of the PTL.

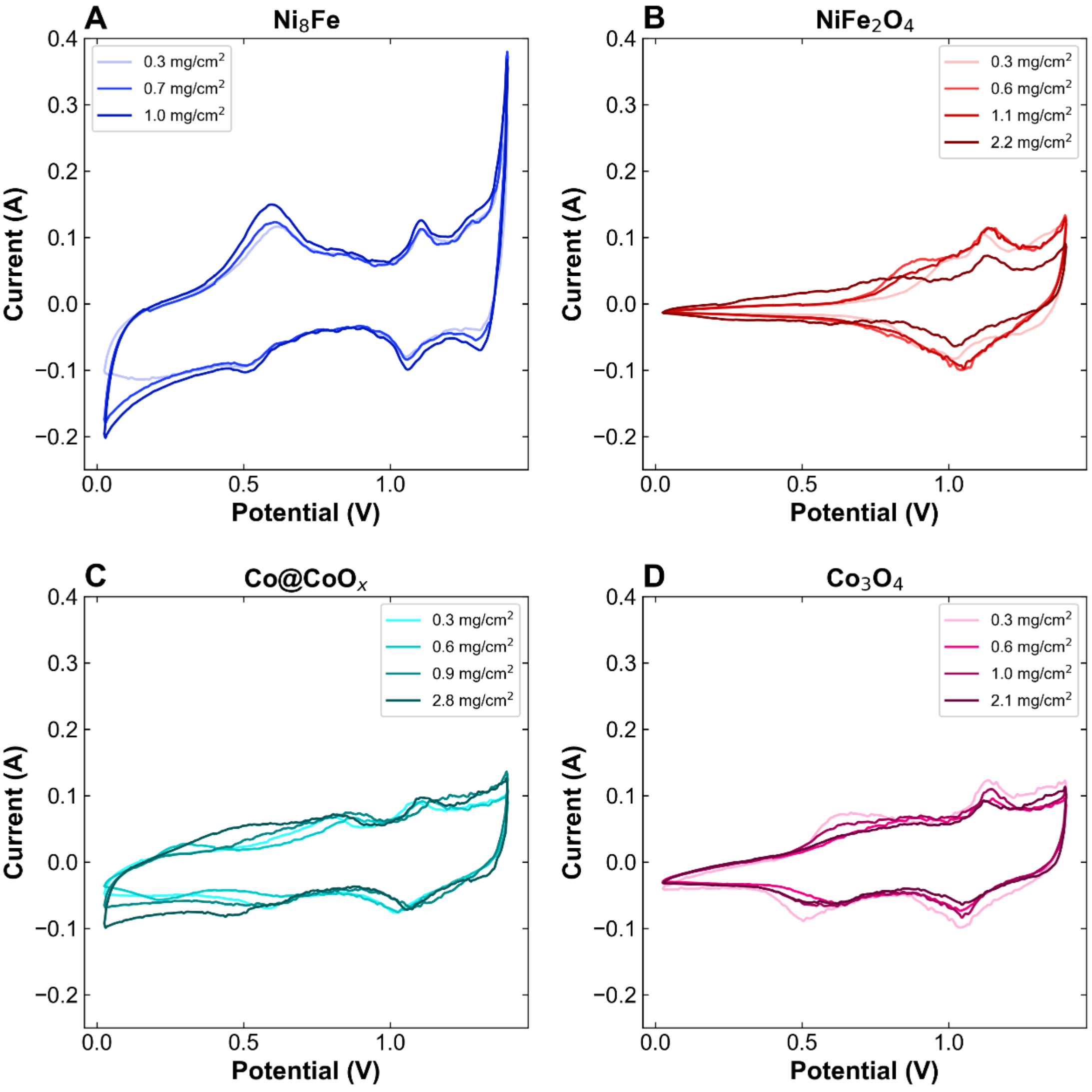

Figure S16. Cyclic voltammograms for various loadings of (**A**) Ni_8Fe (blue), (**B**) $NiFe_2O_4$ (red), (**C**) Co@CoO_x (teal), and (**D**) Co_3O_4 (pink) anodes at 100 mV/s. Darker colors correspond to higher loadings.

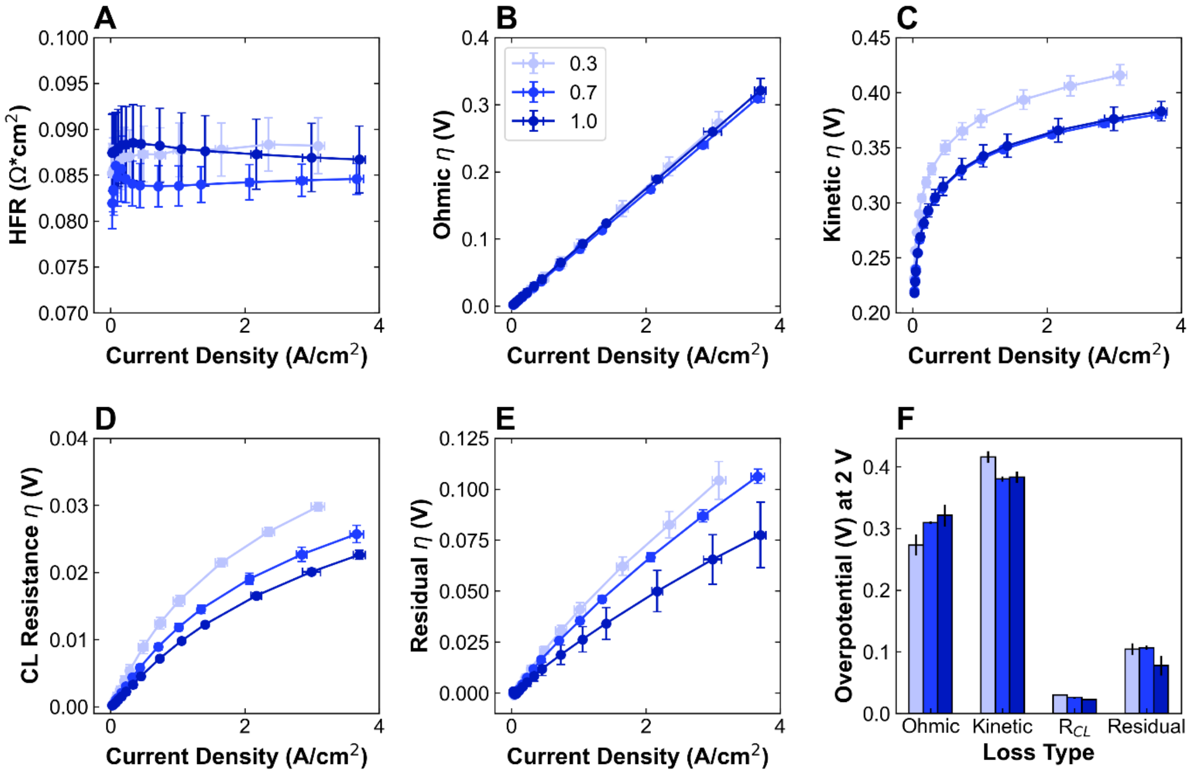

Figure S17. Voltage loss breakdown analysis for Ni_8Fe at 0.3 (light blue), 0.7, and 1.0 (dark blue) mg/cm^2 loadings, from **Figure 5**. As a function of current density: (**A**) HFR calculated from EIS and overpotentials due to (**B**) ohmic, (**C**) kinetic, (**D**) catalyst layer resistance, and (**E**) residual losses. (**F**) Summary of the distribution of overpotential between the different voltage losses at 2 V. Data is reported in triplicate. Darker colors correspond to higher loadings.

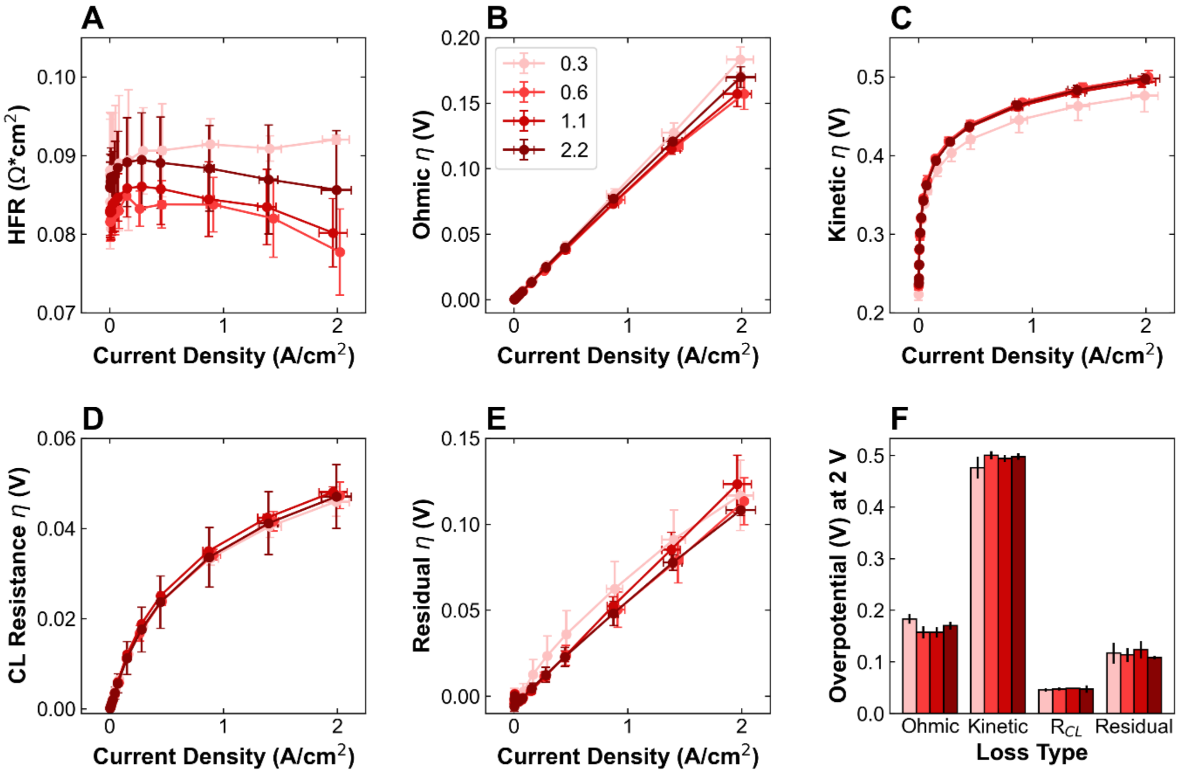

Figure S18. Voltage loss breakdown analysis for $NiFe_2O_4$ at 0.3 (light red), 0.6, 1.1, and 2.2 (dark red) mg/cm^2 loadings, from **Figure 5**. As a function of current density: (**A**) HFR calculated from EIS and overpotentials due to (**B**) ohmic, (**C**) kinetic, (**D**) catalyst layer resistance, and (**E**) residual losses. (**F**) Summary of the distribution of overpotential between the different voltage losses at 2 V. Data is reported in triplicate. Darker colors correspond to higher loadings.

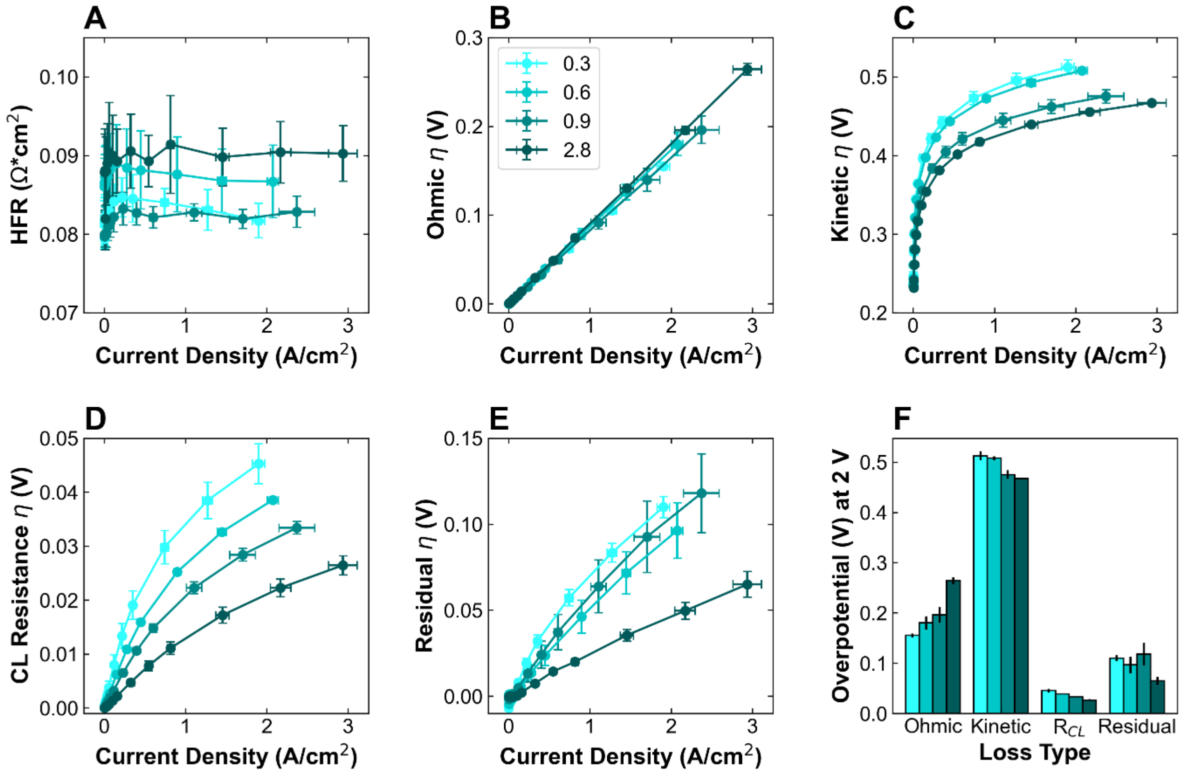

Figure S19. Voltage loss breakdown analysis for Co@CoO_x at 0.3 (turquoise), 0.6, 0.9, and 2.8 (dark teal) mg/cm^2 loadings, from **Figure 5**. As a function of current density: (**A**) HFR calculated from EIS and overpotentials due to (**B**) ohmic, (**C**) kinetic, (**D**) catalyst layer resistance, and (**E**) residual losses. (**F**) Summary of the distribution of overpotential between the different voltage losses at 2 V. Data is reported in triplicate. Darker colors correspond to higher loadings.

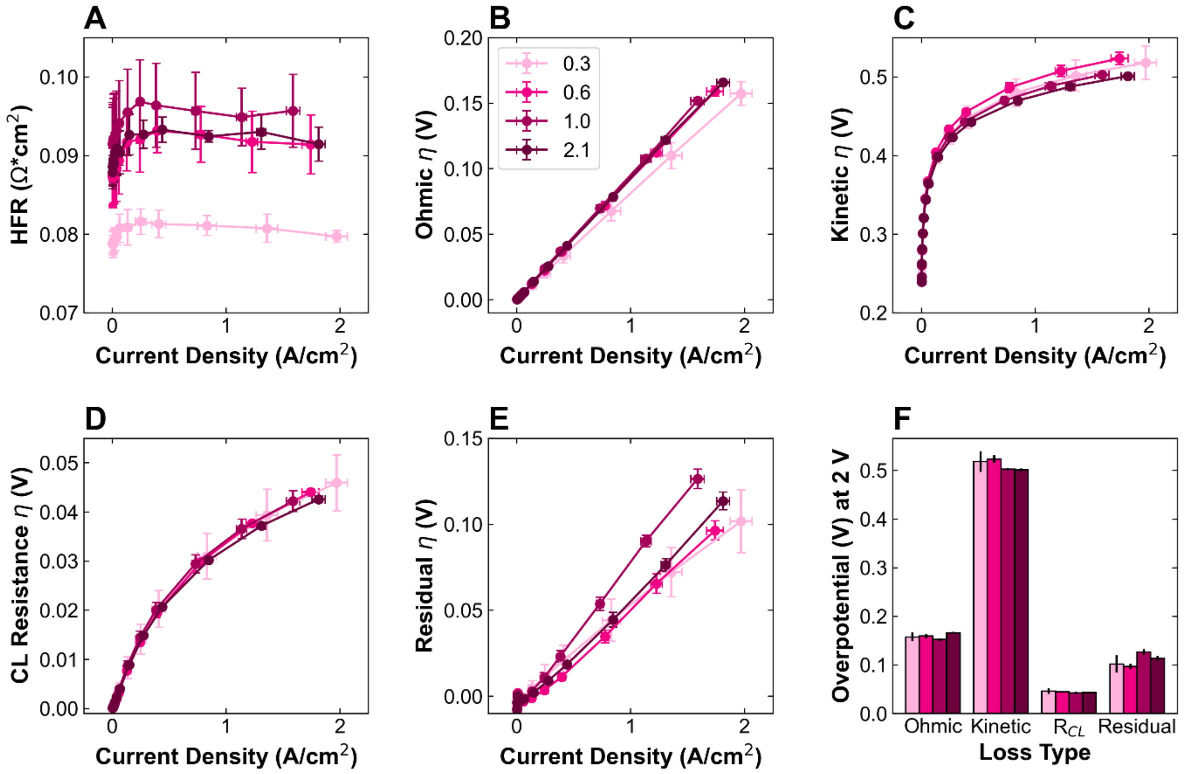

Figure S20. Voltage loss breakdown analysis for Co_3O_4 at 0.3 (light pink), 0.6, 1.0, and 2.1 (dark pink) mg/cm^2 loadings, from **Figure 5**. As a function of current density: (**A**) HFR calculated from EIS and overpotentials due to (**B**) ohmic, (**C**) kinetic, (**D**) catalyst layer resistance, and (**E**) residual losses. (**F**) Summary of the distribution of overpotential between the different voltage losses at 2 V. Data is reported in triplicate. Darker colors correspond to higher loadings.

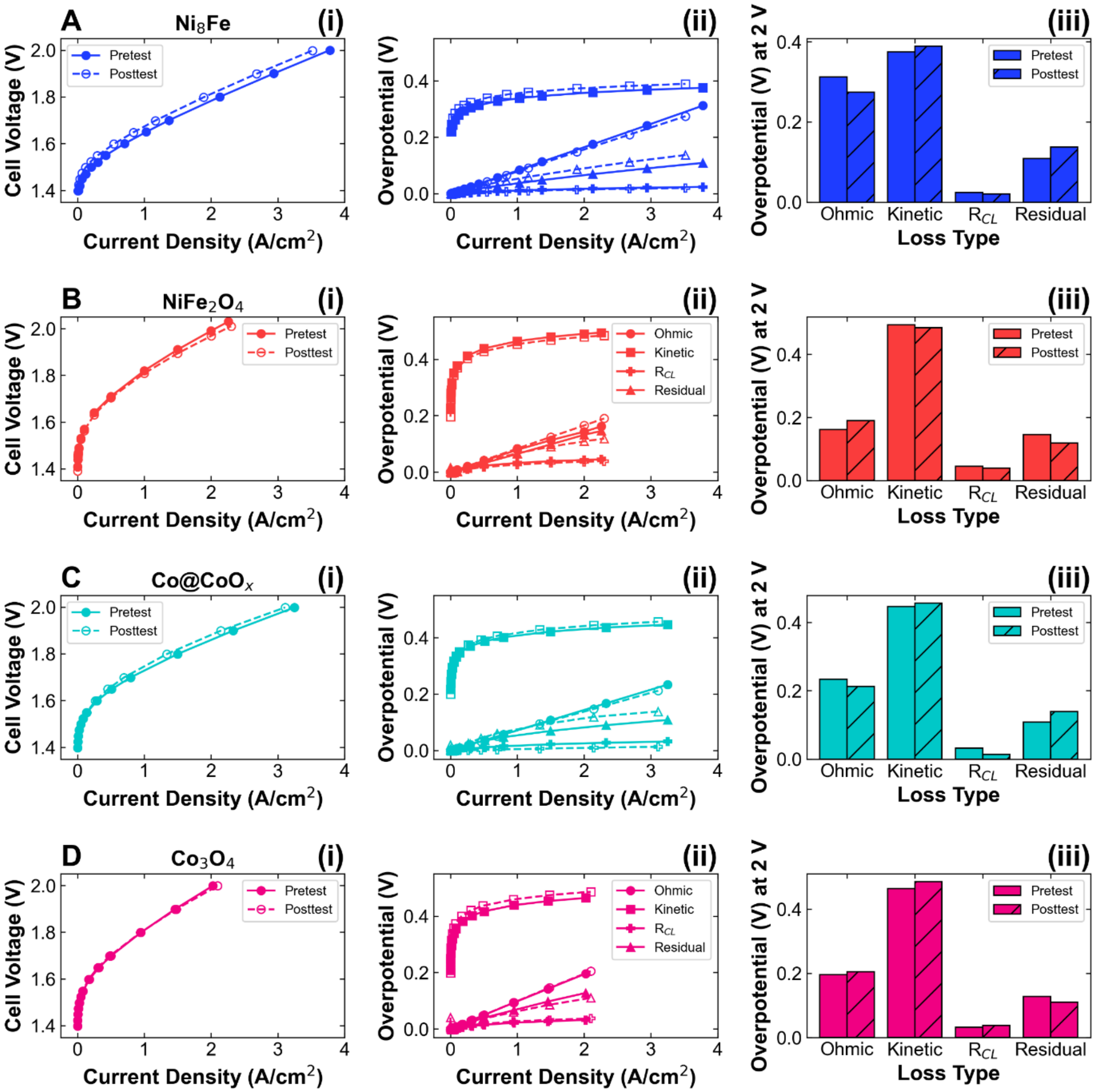

Figure S21. AEMWE performance before and after durability testing at 2 V for (**A**) Ni_8Fe (blue, 50 h), (**B**) $NiFe_2O_4$ (red, 16 h), (**C**) Co@CoO_x (teal, 30 h), and (**D**) Co_3O_4 (pink, 35 h) at ~0.6 mg/cm^2 loading. (**i**) Polarization curves, (**ii**) ohmic (circles), kinetic (squares), catalyst layer resistance (pluses), and residual (triangles) overpotentials, and (**iii**) summary of the distribution of overpotential between the different voltage losses at 2 V. In (**i-ii**), pretest data is denoted by solid lines and filled symbols, while posttest data is denoted by dashed lines and open symbols. In (**iii**), pretest data has solid bars and posttest data has diagonal lines through the bars.

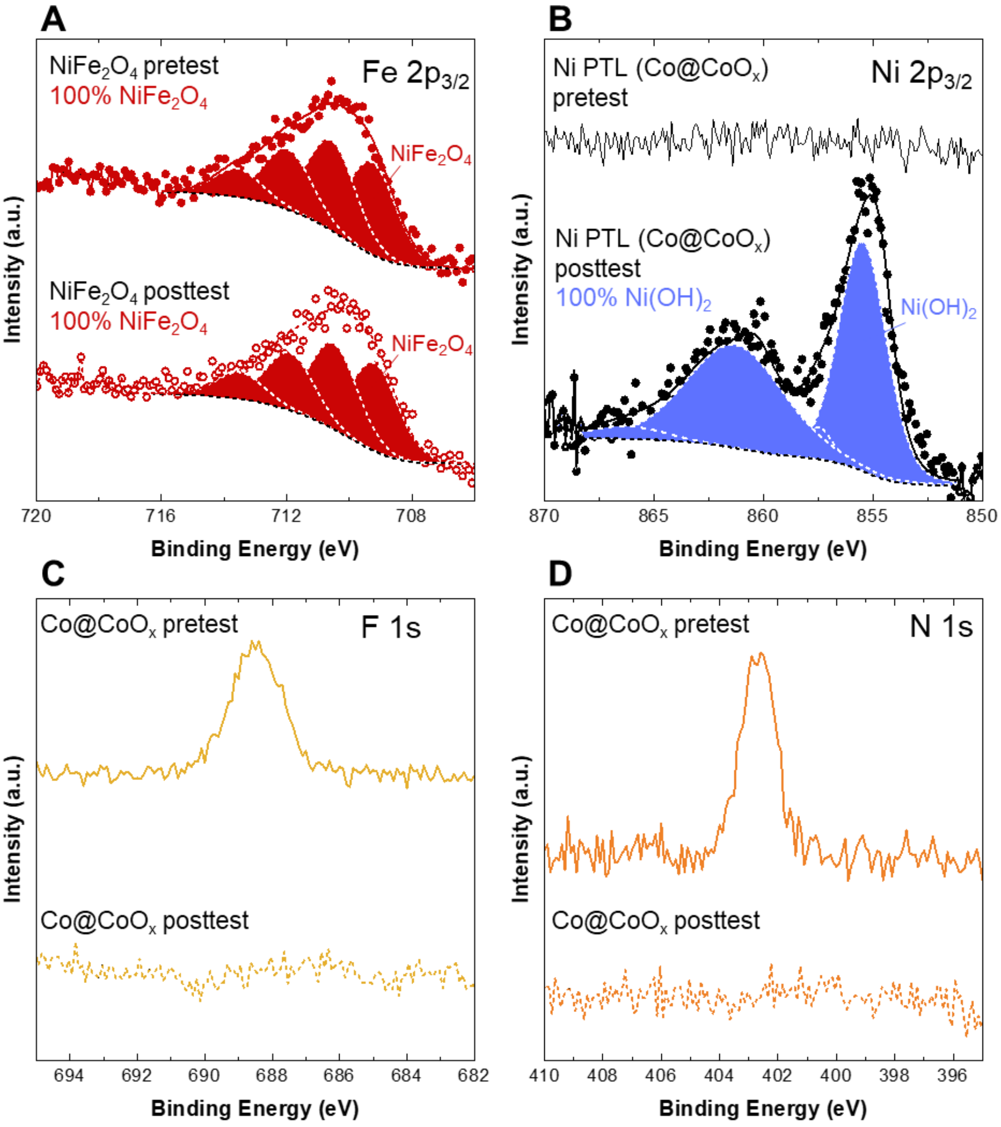

Figure S22. (**A**) Fe $2p_{3/2}$ XPS spectra for $NiFe_2O_4$ (1.1 mg/cm^2) before and after chronoamperometry at 2 V for 12 h, showing fit to $NiFe_2O_4$. (**B**) Ni $2p_{3/2}$ XPS spectra for Co@CoO_x (0.3 mg/cm^2 loading) before and after chronopotentiometry at 2 V for 9 h, showing lower catalyst coverage after testing such that there is significant signal for the Ni PTL. The PTL fits well to $Ni(OH)_2$, indicating that its surface is oxidized. (**C**) F 1s and (**D**) N 1s spectra for Co@CoO_x before and after chronoamperometry at 2 V for 9 h, showing the loss of ionomer after testing.

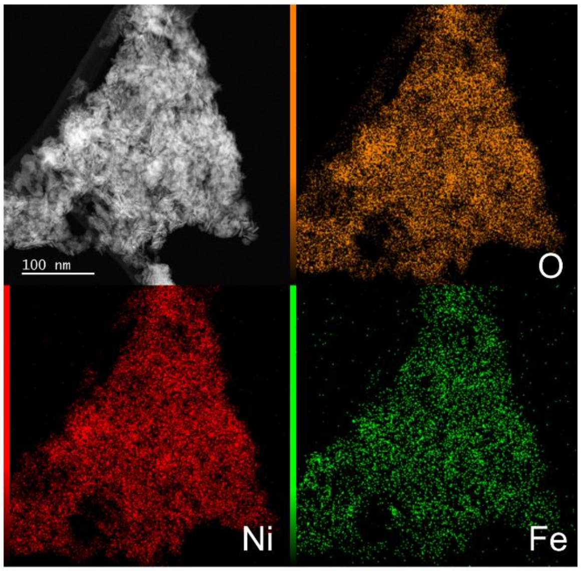

Figure S23. HAADF-STEM image and O (orange), Ni (red), and Fe (green) EDS maps for Ni_8Fe (1.0 mg/cm^2 loading) after chronopotentiometry at 1 A/cm^2 for 110 h.

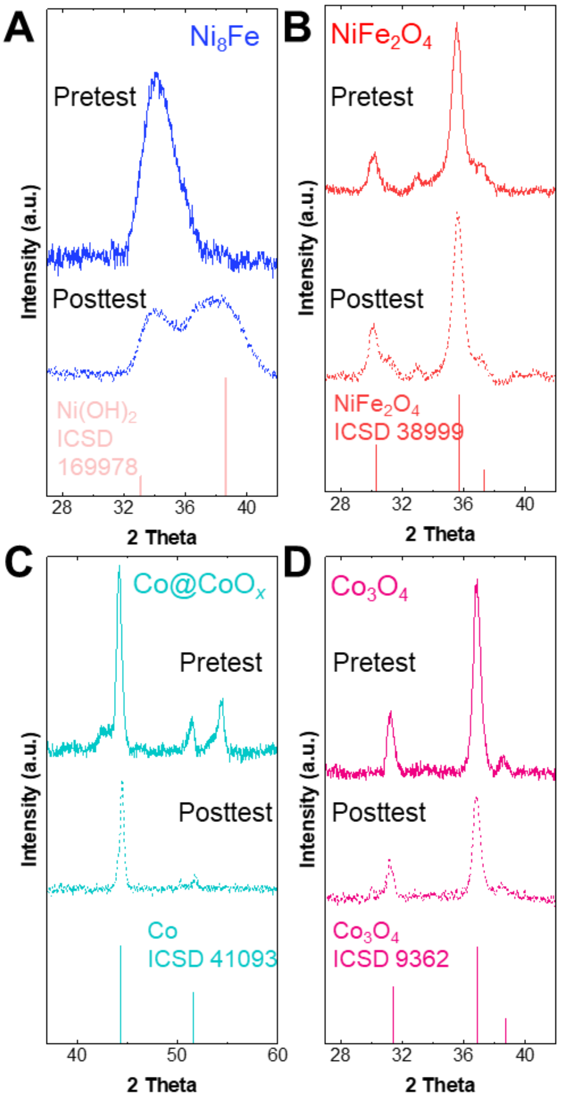

Figure S24. XRD patterns pretest (solid lines) and posttest (dashed lines) for (**A**) Ni_8Fe (blue, 1 mg/cm^2, 110 h at 1 A/cm^2), (**B**) $NiFe_2O_4$ (red, 1.1 mg/cm^2, 12 h at 2 V), (**C**) Co@CoO_x (teal, 0.6 mg/cm^2, 30 h at 2 V), and (**D**) Co_3O_4 (pink, 0.6 mg/cm^2, 35 h at 2 V). 2-Theta ranges are restricted for (**A, B**) to avoid interference with the Ni PTL. For (**C, D**), samples were scraped off of the PTL to avoid interference with the Ni PTL. ICSD references for $Ni(OH)_2$ (pink, 169978), $NiFe_2O_4$ (red, 38999), Co (teal, 41093), and Co_3O_4 (pink, 9362) are given as stick patterns.

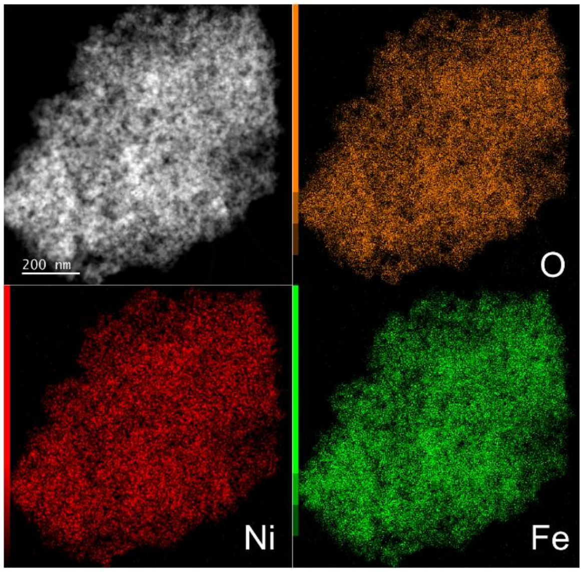

Figure S25. HAADF-STEM image and O (orange), Ni (red), and Fe (green) EDS maps for $NiFe_2O_4$ after testing (1 mg/cm^2, 12 h at 2 V).

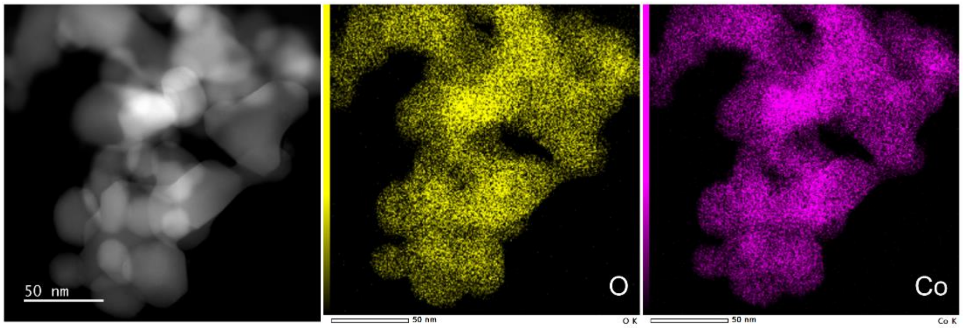

Figure S26. HAADF-STEM image and O (yellow) and Co (pink) EDS maps for Co_3O_4 after testing (0.6 mg/cm^2 loading, 35 h at 2 V).

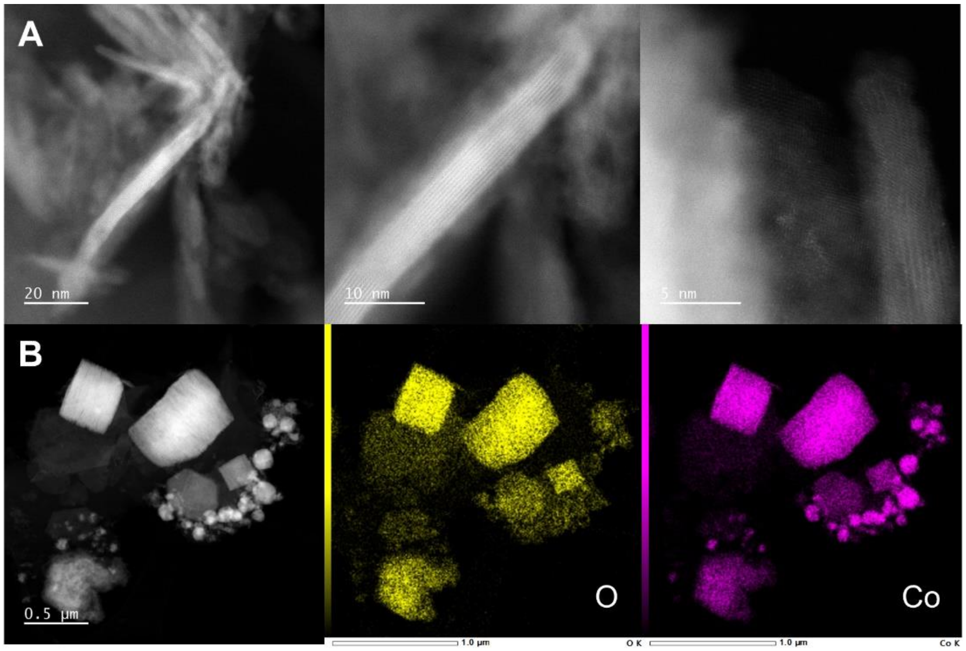

Figure S27. (**A**) HAADF-STEM images of needle-like structures and (**B**) HAADF-STEM images and O (yellow) and Co (pink) EDS maps for Co@CoO$_x$ after testing (0.6 mg/cm^2 loading, 30 h at 2 V). Some particles retain the core-shell structure, while others are completely oxidized.

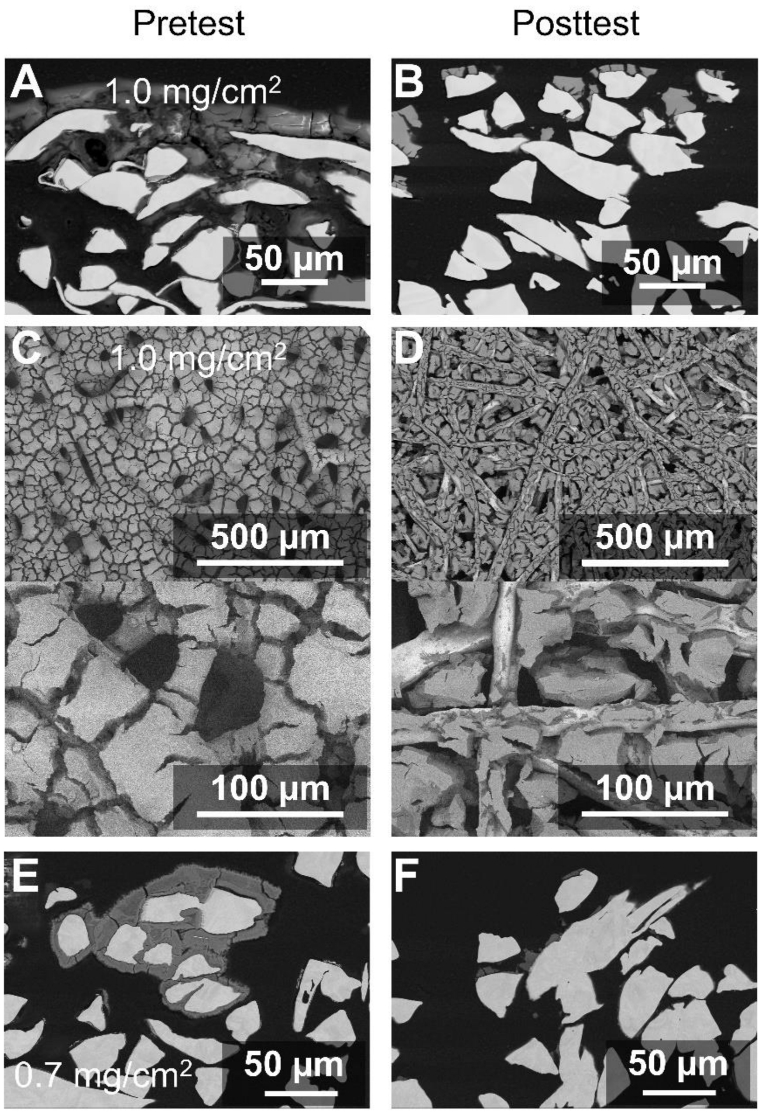

Figure S28. SEM images of Ni_8Fe catalyst layers. Cross-section images of 1.0 mg/cm^2 loading on Ni PTL (**A**) pretest and (**B**) posttest (110 h at 1 A/cm^2). Top-down images of 1.0 mg/cm^2 loading on Ni PTL (**C**) pretest and (**D**) posttest (110 h at 1 A/cm^2). Cross-section images of 0.7 mg/cm^2 loading on Ni PTL (**E**) pretest and (**F**) posttest (48 h at 2 V).

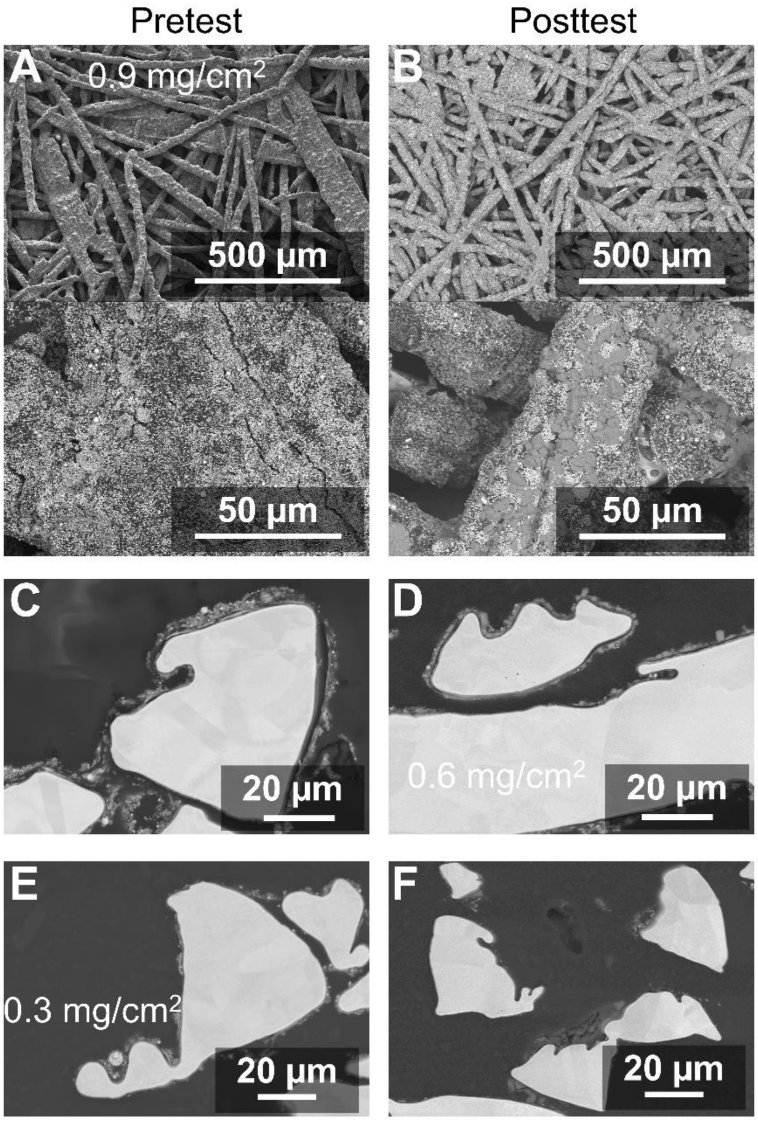

Figure S29. SEM images of Co@CoO$_x$ catalyst layers. Top-down images of 0.9 mg/cm^2 loading on Ni PTL (**A**) pretest and (**B**) posttest (short-term activity testing). Cross-section images of 0.6 mg/cm^2 loading on Ni PTL (**C**) pretest and (**D**) posttest (30 h at 2 V). Cross-section images of 0.3 mg/cm^2 loading on Ni PTL (**E**) pretest and (**F**) posttest (11 h at 2 V).

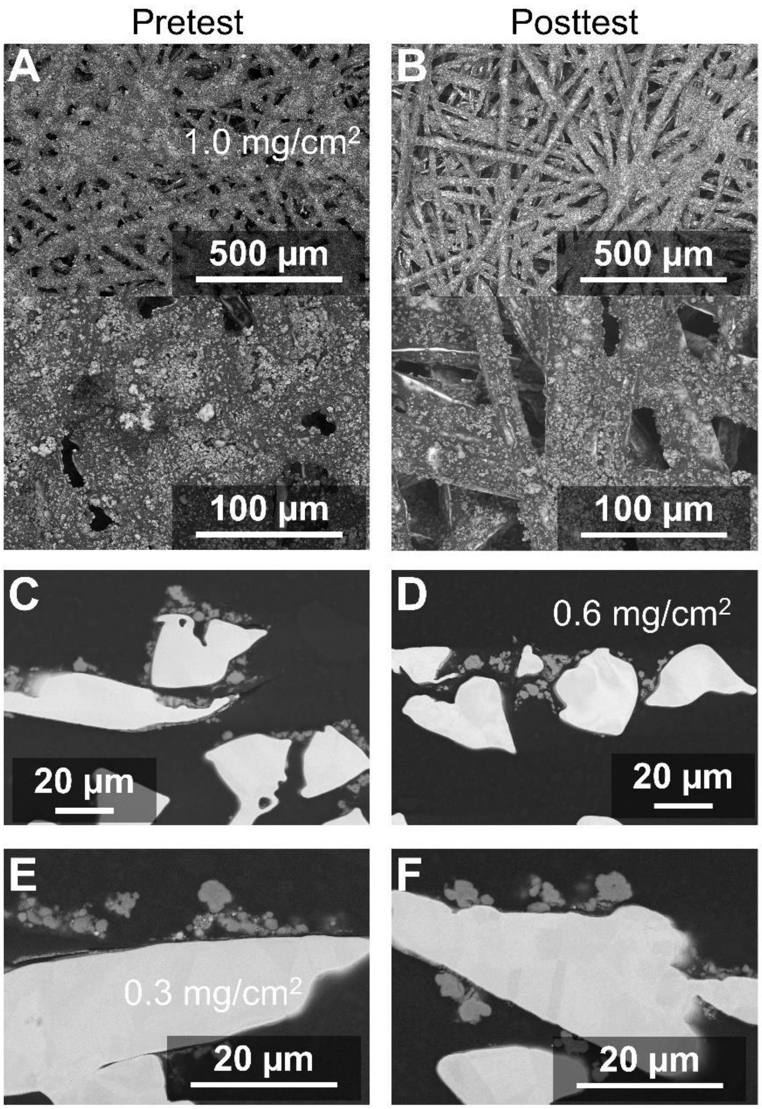

Figure S30. SEM images of Co_3O_4 catalyst layers. Top-down images of 1.0 mg/cm^2 loading on Ni PTL (**A**) pretest and (**B**) posttest (short-term activity testing). Cross-section images of 0.6 mg/cm^2 loading on Ni PTL (**C**) pretest and (**D**) posttest (35 h at 2 V). (**E**) Cross-section and top-down images of 0.3 mg/cm^2 loading on Ni PTL pretest and (**F**) cross-section posttest (18 h at 2 V).

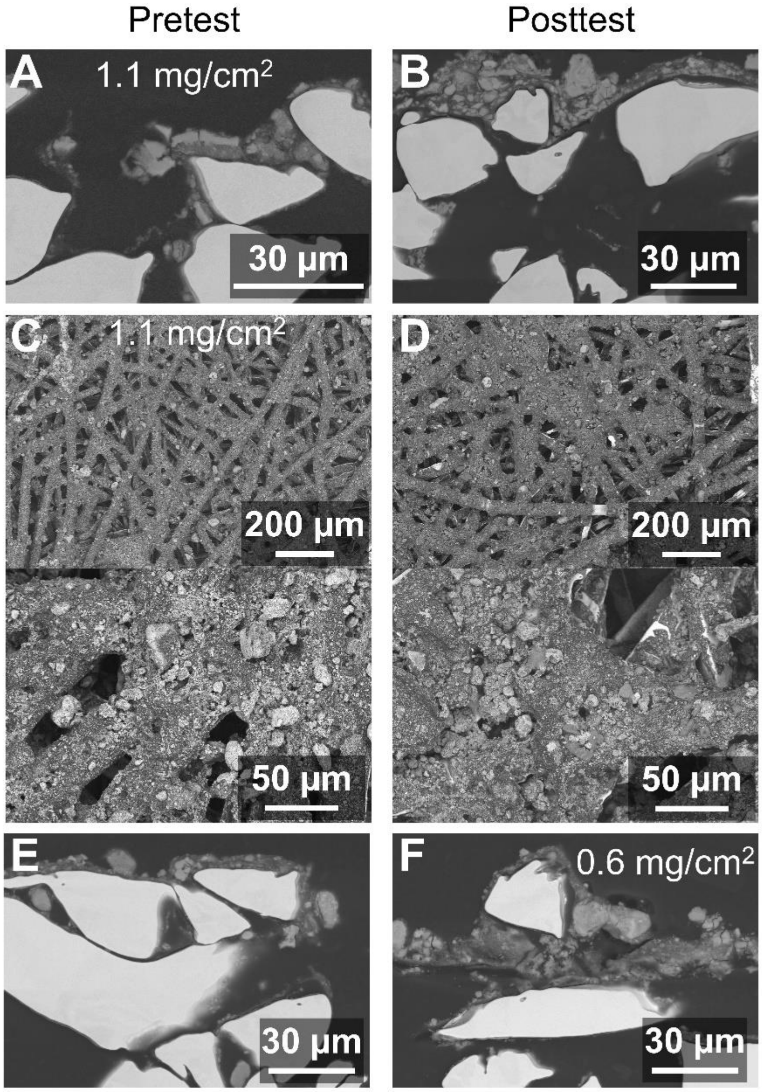

Figure S31. SEM images of $NiFe_2O_4$ catalyst layers. Cross-section images of 1.1 mg/cm^2 loading on Ni PTL (**A**) pretest and (**B**) posttest (13 h at 2 V). Top-down images of 1.1 mg/cm^2 loading on Ni PTL (**C**) pretest and (**D**) posttest (13 h at 2V). Cross-section images of 0.6 mg/cm^2 loading on Ni PTL (**E**) pretest and (**F**) posttest (14 h at 2 V).

References

(1) Balej, J. Water Vapour Partial Pressures and Water Activities in Potassium and Sodium Hydroxide Solutions over Wide Concentration and Temperature Ranges. *Int. J. Hydrog. Energy* **1985**, *10* (4), 233–243. https://doi.org/10.1016/0360-3199(85)90093-X.

(2) Ursúa, A.; Sanchis, P. Static–Dynamic Modelling of the Electrical Behaviour of a Commercial Advanced Alkaline Water Electrolyser. *Int. J. Hydrog. Energy* **2012**, *37* (24), 18598–18614. https://doi.org/10.1016/j.ijhydene.2012.09.125.

(3) Padgett, E.; Bender, G.; Haug, A.; Lewinski, K.; Sun, F.; Yu, H.; Cullen, D. A.; Steinbach, A. J.; Alia, S. M. Catalyst Layer Resistance and Utilization in PEM Electrolysis. *J. Electrochem. Soc.* **2023**, *170* (8), 084512. https://doi.org/10.1149/1945-7111/acee25.

(4) Biesinger, M. C.; Payne, B. P.; Grosvenor, A. P.; Lau, L. W. M.; Gerson, A. R.; Smart, R. St. C. Resolving Surface Chemical States in XPS Analysis of First Row Transition Metals, Oxides and Hydroxides: Cr, Mn, Fe, Co and Ni. *Appl. Surf. Sci.* **2011**, *257* (7), 2717–2730. https://doi.org/10.1016/j.apsusc.2010.10.051.
